# A facile and scalable in production non-viral gene engineered mesenchymal stem cells for effective suppression of temozolomide-resistant (TMZR) glioblastoma growth

**DOI:** 10.1186/s13287-020-01899-x

**Published:** 2020-09-11

**Authors:** Geraldine Xue En Tu, Yoon Khei Ho, Zhi Xu Ng, Ke Jia Teo, Tseng Tsai Yeo, Heng-Phon Too

**Affiliations:** 1 Department of Biochemistry, National University of Singapore, Singapore, 117596 Singapore; 2 Division of Neurosurgery, Department of General Surgery, Khoo Teck Puat Hospital, Singapore, 768828 Singapore; 3 Division of Neurosurgery, Department of General Surgery, National University Hospital, National University Health Systems, Singapore, Singapore

**Keywords:** Temozolomide-resistant glioma, Mesenchymal stem cells, Transfection, Prodrug therapy

## Abstract

**Background:**

Mesenchymal stem cells (MSCs) serve as an attractive vehicle for cell-directed enzyme prodrug therapy (CDEPT) due to their unique tumour-nesting ability. Such approach holds high therapeutic potential for treating solid tumours including glioblastoma multiforme (GBM), a devastating disease with limited effective treatment options. Currently, it is a common practice in research and clinical manufacturing to use viruses to deliver therapeutic genes into MSCs. However, this is limited by the inherent issues of safety, high cost and demanding manufacturing processes. The aim of this study is to identify a facile, scalable in production and highly efficient non-viral method to transiently engineer MSCs for prolonged and exceptionally high expression of a fused transgene: yeast cytosine deaminase::uracil phosphoribosyl-transferase::green fluorescent protein (CD::UPRT::GFP).

**Methods:**

MSCs were transfected with linear polyethylenimine using a cpg-free plasmid encoding the transgene in the presence of a combination of fusogenic lipids and β tubulin deacetylase inhibitor (Enhancer). Process scalability was evaluated in various planar vessels and microcarrier-based bioreactor. The transfection efficiency was determined with flow cytometry, and the therapeutic efficacy of CD::UPRT::GFP expressing MSCs was evaluated in cocultures with temozolomide (TMZ)-sensitive or TMZ-resistant human glioblastoma cell lines. In the presence of 5-fluorocytosine (5FC), the 5-fluorouracil-mediated cytotoxicity was determined by performing colometric MTS assay. In vivo antitumor effects were examined by local injection into subcutaneous TMZ-resistant tumors implanted in the athymic nude mice.

**Results:**

At > 90% transfection efficiency, the phenotype, differentiation potential and tumour tropism of MSCs were unaltered. High reproducibility was observed in all scales of transfection. The therapeutically modified MSCs displayed strong cytotoxicity towards both TMZ-sensitive and TMZ-resistant U251-MG and U87-MG cell lines only in the presence of 5FC. The effectiveness of this approach was further validated with other well-characterized and clinically annotated patient-derived GBM cells. Additionally, a long-term suppression (> 30 days) of the growth of a subcutaneous TMZ-resistant U-251MG tumour was demonstrated.

**Conclusions:**

Collectively, this highly efficient non-viral workflow could potentially enable the scalable translation of therapeutically engineered MSC for the treatment of TMZ-resistant GBM and other applications beyond the scope of this study.

## Background

Glioblastoma multiforme (GBM) is the most aggressive malignant brain tumour and is extremely difficult to treat. The current standard of care involves surgical resection, followed by radiation and temozolomide (TMZ) treatment. Despite the vigorous treatment regime, almost all patients relapse [1, 2]. Previous studies have shown that only 30–40% of GBMs respond favourably to TMZ treatment [3–5]. Furthermore, patients who are responsive to TMZ treatment rapidly gain resistance resulting in the development of recurrence where survival is typically measured in months [3, 6]. There is presently no standard second-line chemotherapy available for TMZ-resistant GBM as systemic treatment options are limited by poor response. Such treatment failure is largely attributed to the invasive nature of GBM, ineffective delivery of chemo-drugs across the blood-brain barrier and associated dose-limiting systemic toxicities [7–10]. Thus, tumour targeting strategies to achieve high local concentration of cytotoxic agent are critical to improve clinical outcome for TMZ-resistant GBM.

Owing to their immune-privilege and tumour-nesting properties, mesenchymal stem cells (MSCs) have been explored as cellular vehicles for cell-directed enzyme prodrug therapy (CDEPT) in targeting GBM [11–15]. CDEPT is one of the safer alternatives to conventional chemotherapy as it overcomes dose-limiting toxicities by enabling maximum cytotoxic effect at the vicinity of tumours through the in situ conversion of a non-toxic prodrug into active drug [16, 17]. Proof-of-concept and safety was reported in a first-in-human study (NCT01172964) where a neural stem cell (NSC)-mediated cytosine deaminase/5-fluorocytosine (CD/5FC) prodrug system was tested in patients with recurrent GBM [18]. CD converts a non-toxic prodrug (5FC) into an anticancer drug 5-fluorouracil (5FU) that is able to exert a potent bystander effect through simple diffusion into the neighbouring cells without direct cell-to-cell contact [19, 20]. Particularly in GBM, the bystander effect of prodrug therapy has been demonstrated in both in vitro and in vivo model [21, 22].

Following the report that demonstrated promising treatment of intracranial rat GBM with the use of CD expressing NSCs [23], multiple in vivo animal studies have shown therapeutic efficacy with NSCs [24–27] and MSCs [13, 28–30]. Interestingly, a complete regression of GBM was previously reported by the administration of MSCs expressing a fusion transgene of uracil phosphoribosyl-transferase (UPRT) to CD (CD::UPRT) into the brain tissue at the postoperative resection cavity [13]. UPRT directly converts 5FU to 5-fluorodeoxyuridine monophosphate and has been shown to significantly improve antitumoural activity of CD/5FC system [31, 32]. Pre-clinical studies have demonstrated the therapeutic potential of CDEPT in GBM cell lines such as C6 [13, 28–30] as well as U-251MG and U-87MG, [33] which are TMZ sensitive [3, 34]. However, the cytotoxic effect of such strategy in targeting TMZ-resistant GBM has yet to be investigated. This study aims to acquire greater understanding of the clinical utility of MSC-mediated CDEPT in TMZ-resistant GBM treatment.

Currently, most if not all methods to modify MSCs use viruses to deliver genes due to the lack of non-viral alternatives [35–37]. Although non-viral gene delivery methods are safer and more flexible than viral methods, they are often less efficient (~ 2–50% transfection efficiency) [35, 38, 39] and can be toxic to MSCs [40–42]. By mitigating intracellular trafficking barriers of cationic polymer gene delivery, we previously reported the high transfection efficiency of MSCs without the use of viruses. However, the protocol was limited by its scalability due to the necessity of a low-speed centrifugation step [43, 44]. In the present study, we developed a facile, rapid and scalable transfection protocol to generate MSCs producing CD::UPRT::GFP. Without the need for antibiotic selection, the quality of the engineered cells satisfied the release criteria reported in recent CDEPT clinical trials [18, 45]. To be more clinically relevant, this study examined the cytotoxic effects of this non-viral engineered MSCs on TMZ-sensitive and TMZ-resistant GBM as well as the uniquely characterized and clinically annotated GBM cell lines derived from patient surgical samples. The utility of these cells was also demonstrated in a tumour model using TMZ-resistant GBM cells.

## Methods

### Cell culture

Human adipose-derived mesenchymal stem cells (AD-MSC, RoosterBio), primary human dermal fibroblast (ATCC, PCS-201-012) and U-87MG cell line (ATCC, HTB-14) were cultured and maintained according to the manufacturer’s instruction. U-251MG cell line (U-251MG) was kindly provided by Paula Lam (Duke NUS Medical School). U-251MG was cultured in Dulbecco modified Eagle medium (DMEM) supplemented with 10% fetal bovine serum (FBS, Biowest). U-251MG- and U-87MG-resistant cell lines were obtained by progressive adaptation of the parental cells with increasing concentrations of temozolomide (TMZ) as described [46]. Initial concentration of 10 μM was used. Increasing temozolomide resistance concentrations of 10, 20 and 40 μM were developed. In glioma patients, the peak concentrations of temozolomide were predicted to range from 14.9 to 34.5 μM, with 100% bioavailability [47, 48]. Thus, cell lines adapted to 40 μM TMZ (U251-MG™^ZR40^) were selected as the clinically relevant TMZ-resistant cell line. Patient-derived glioma cell lines from HGCC biobank were cultured in DMEM F12/neurobasal media supplemented with B27, N2, EGF and FGF according to the published protocol [49]. Cells were kept at 37 °C in humidified atmosphere and 5% CO_2_.

### Transfection and expression analysis

AD-MSCs were kept at passages 3–7 for transfection. AD-MSCs were seeded at 50,000 per well in 24-well plate format and incubated for 24 h before transfection. Polyethylenimine MAX (PEI, Polyscience) was added to PBS at 1 μg of plasmid DNA to 3 μL of PEI (1 mg/mL). The mixture, at a total volume of 50 μL, was incubated at room temperature for 15 min. The complex was then added to the cell culture in a dropwise manner. Transfection Enhancer were supplemented to complete media before the addition of transfection mixture into the culture. The Enhancer consist of DOPE/CHEMS (9:2 M ratio Fusogenic lipid, Polar Avanti Lipid) and 1.25 μM Vorinostat (Histone deacetylase inhibitor; HDACi, Bio Vision) [43, 44]. The culture media were replaced with fresh media at 24 h post-transfection. Then, cells were further incubated for at least 24 h before analysis.

### Construction of expression plasmid containing fusion of CD:UPRT and GFP

Briefly, the CD::UPRT::GFP plasmid was constructed using the vector backbone of pCpGfree-Lucia (InvivoGen). Lucia was replaced by CD::UPRT and GFP using pSELECT-zeo-FcyFur and CpG-free GFP:Sh as template. The functional plasmid of interest, CD:UPRT:GFP, was constructed using cross-lapping in vitro assembly cloning method as described [50]. The construct (Additional file 1) was transformed using heat shock method with chemically competent *Escherichia coli* GT115 (Invivogen) and propagated under Zeocin antibiotic selection. The plasmids were then extracted and purified with E.Z.N.A. endo-free plasmid maxi kit according to the manufacturer’s instruction (Omega Bio-tek).

### Imaging

Cell images were taken with EVOS FL Cell Imaging System (ThermoFisher Scientific) equipped with fluorescent light cubes for viewing of DAPI (Ex357/Em447) and GFP (Ex470/Em510) fluorescence.

### In vitro cytotoxic studies

#### TMZ and 5FU sensitivity

Quadruplicates of different glioma cell lines were plated in 96-well plates at 2500 cells per well. The following day, culture media were replaced with DMEM supplemented with 2% FBS, with different concentrations of TMZ (0–250 μM) or 5FU (0–500 μg/mL). Five days later, cell viability was measured by MTS assay using CellTiter 96® AQueous One Solution Cell Proliferation Assay (Promega). Total absorbance was measured using Synergy™ H1 Microplate Reader at 490 nm. The percentage of cell viability was calculated with no treatment controls were set at 100%.

#### Coculture experiment

Replicates of different glioma cell lines were plated in 96-well plates, 2500 cells per well. Five hours later, increasing numbers of either non-modified or CD:UPRT:GFP producing MSCs were plated to the cancer cell culture at the MSC to cancer ratios of 1 to 1, 5, 10, 25, 50 and 100. The culture media were replaced with DMEM supplemented with 2% FBS, with or without 5FC (0–200 μg/mL). Seven days later, cell viability was measured by MTS assay. The in vitro cytotoxic study has been reproduced once with 6 biological replicates for each condition.

### Flow cytometry analysis

#### Transfection efficiency

Cells were washed twice with 1XPBS and trypsinized for 5 min. After detachment of cells, complete media were added at 4 times of the volume of trypsin. The suspension cells were subsequently transferred to conical or Eppendorf tubes. Cell pellets were obtained from centrifugation at 300*g* for 3 min. Cells were then resuspended in PBS prior to analysis. Percentage of fluorescence positive MSCs was quantified by Attune NxT Flow Cytometer system (ThermoFisher Scientific), and the raw data was analysed using non-modified MSCs as negative controls at < 0.5%, using Invitrogen Attune NxT software (ThermoFisher Scientific).

#### Cell cycle analysis

Parental and TMZ-resistant glioma cell lines were fixed using 70% ethanol in cold for at least 2 h. Cell pellets were obtained upon centrifugation at 300×*g* for 3 min. Cells were stained using 0.1% Triton X-100, 0.2 mg/mL RNase A and 20 μg/mL propidium iodide (PI) in 1XPBS for 15 min before FACS analysis. Cell cycle distribution of parental and TMZ-resistant cell line were analysed to obtain the percentage of population in G0/G1, S and G2/M phases.

#### Phenotypic characterization

To examine the phenotype of CD:UPRT:GFP producing AD-MSC, cells were labelled with MSC Phenotyping Kit consisting of antibodies CD73, CD105, CD14, CD20, CD34, CD45 and HLA-DR (Miltenyi Biotech) according to the manufacturer’s instructions. CD90-PE (Miltenyi Biotech) was used separately for CD90 expression characterization. Expression of the markers were analysed using isotype controls with FACS.

### Differentiation potential of CD:UPRT:GFP producing AD-MSC

Differentiation of AD-MSC was induced with StemPr Osteogenesis Differentiation Kit and StemPro adipogenesis (ThermoFisher Scientific). Briefly, osteogenesis and adipogenesis were induced by culturing MSCs with differentiation media for 21 and 14 days, respectively. Alizarin red S was used for staining for calcium deposits for osteogenic differentiation, and Oil Red O was used for staining for lipid droplets for adipogenic differentiation.

### Cell migration assay

The tumour tropism of AD-MSC was determined using 8.0 μm Transwell (Corning). Glioma cancer cell lines or human fibroblast cells (200,000 cells) in complete media were seeded in the lower chamber of the 24-well plate. After 24-h incubation, the target cells were washed twice with 1xPBS and replaced with serum-free DMEM. Then, 50,000 of non-modified AD-MSCs and/or CD:UPRT:GFP_AD-MSCs (transfected 1 day before indirect coculture) in serum-free media were added into the upper chambers. MSCs that did not migrate were removed from the upper chamber 24 h later. Migrated MSCs were fixed with 4% paraformaldehyde (4% PFA) and stained with 10 μg/mL Hoechst 33342 (ThermoFisher Scientific). Images were taken with EVOS FL Cell Imaging System (ThermoFisher Scientific) at × 10 magnification. The number of migrated cells was counted based on 3 frames per well using ImageJ. This experiment has been reproduced once.

### In vivo cytotoxic effect of non-viral modified CD::UPRT::GFP_AD-MSC/5-FC

The use of animals in the present study was approved by the Institutional Animal Care and Use Committee (IACUC, R18-1383) of the National University of Singapore, and all procedures were carried out in accordance with institutional guidelines. Female athymic nude mice at 5–6-week-old (InVivos) were purchased. To develop glioma subcutaneous models, 5 × 10^6^ U251-MG™^ZR40^ in 50 μL of serum-free DMEM were mixed with Matrigel (Corning, cat no 354234) at a volume ratio of 1:1 and injected subcutaneously. For treatment, either non-modified or CD::UPRT::GFP producing MSCs in 50 μL of serum-free DMEM were injected intratumourally at one site of the tumour bulk when it reaches 100–150 mm^3^. Injections were made perpendicular to the tumour bulk. The vector was injected slowly slowly over ~ 5 s and leave the needle in place for 5 s before removing. One day post-MSC administration, mice were treated intraperitoneally with 500 mg/kg/day of 5FC for four consecutive days. Tumour growth and the weight of mice was monitored every 2–3 days post-treatment, tumour volume was measured with a vernier calliper and calculated according to the formula as follows:

$$ \mathrm{Volume}=\frac{\mathrm{length}\times {\mathrm{width}}^2}{2} $$

The current experiment has been reproduced with a small number of mice in each group (*n* = 3).

### Statistical analysis

Where Student’s *t* test was used, an unpaired two-tailed test was used, with the assumption that changes in the readout are normally distributed.

## Results

### GBM cell lines were sensitive to 5FU treatment regardless of TMZ sensitivity

**Fig. 1:**
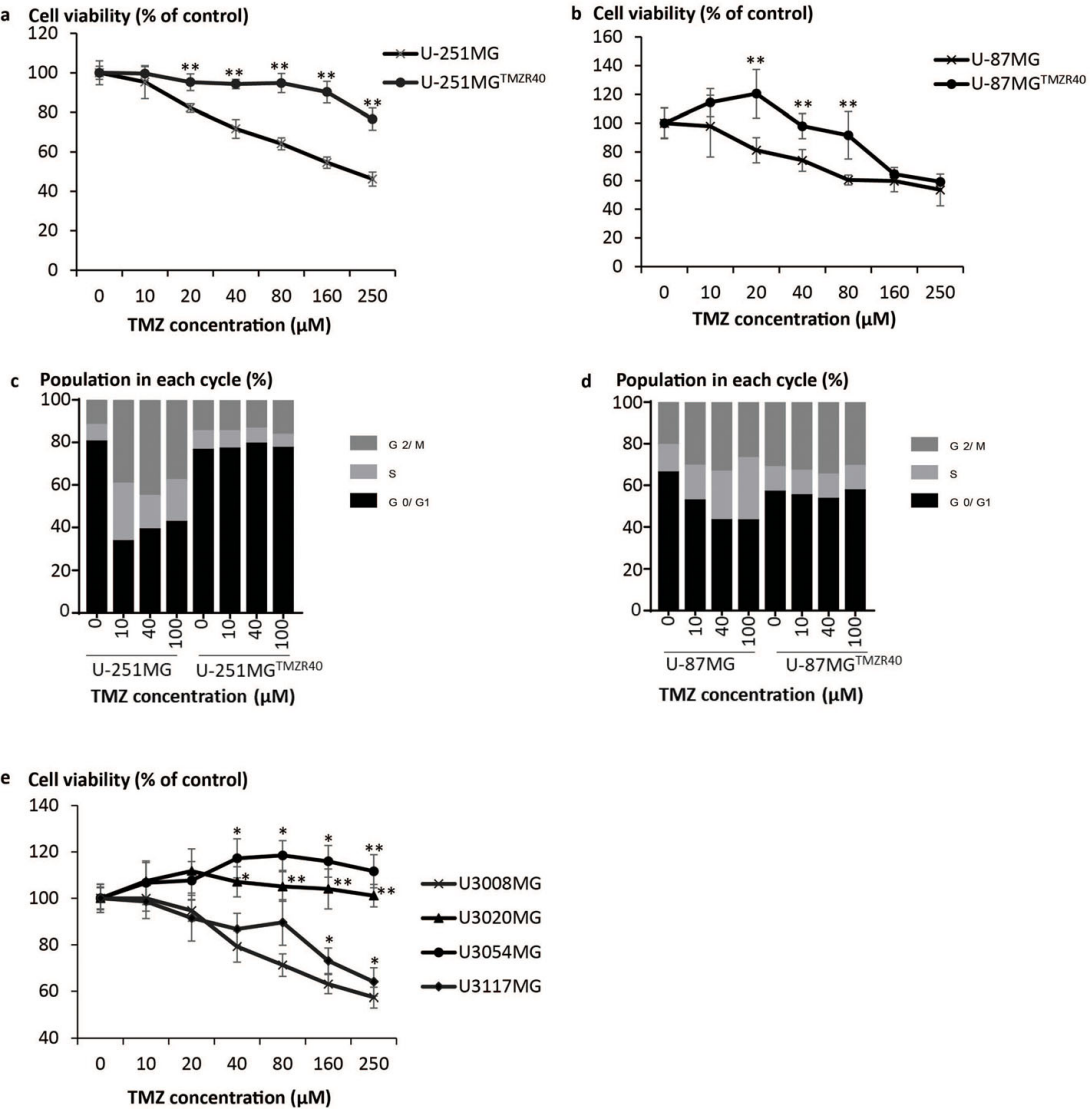
Establishment of TMZR cell models. The TMZ sensitivities of the parental and TMZ adapted **a** U-251MG and **b** U-87MG were determined. Cells cultured in 96-well plate were treated with 0–250 μM TMZ in DMEM media supplemented with 2% FBS. Five days later, cell viability was evaluated spectrophotometrically by standard MTS assay. Cell viability (%) was defined as sample/control × 100%. Conditions without TMZ treatment served as controls. Data of biological quadruplicates were expressed as mean + SD. Significant differences in TMZ sensitivities between parental and TMZR cell lines were calculated using two-tailed Student’s *t* test. **P < 0.01. Cell cycle profile of **c** U-251MG and **d** U-87MG parental and their respective TMZR cell lines were verified by flow cytometry using PI-stained nuclei. The percentage of cells in G1, S and G2 phases is shown. **e** Similar experiment setup was performed to examine TMZ sensitivities of the patient-derived cell lines. The cell viability (%) was calculated accordingly. Significant differences in TMZ sensitivities between U3008MG and other cell lines were calculated using two-tailed Student’s *t* test. *P < 0.05; **P < 0.005

To determine the therapeutic potential of CD::UPRT::GFP_AD-MSC/5FC system in targeting TMZ-resistant (TMZR) GBM, we first established the relevant cell models in culture. TMZ-resistant U-251MG and U-87MG cell lines were developed by the progressive adaptation with increasing concentrations of TMZ [46]. In glioma patients, the peak concentrations of TMZ are thought to be in the range of 14.9–34.5 μM [47]. Accordingly, the TMZ-resistant cell models (U-251MG™^ZR40^ and U-87MG™^ZR40^), insensitive to culture supplemented with 40 μM TMZ, were used for studies described below. Upon TMZ adaptation, the cell viability of U-251MG™^ZR40^ and U-87MG™^ZR40^ cell lines were not affected by exposure to 10, 20 or 40 μM TMZ (Fig. 1a, b). Interestingly, U-251MG™^ZR40^ displayed higher resistance to TMZ than U-87MG™^ZR40^ where reduction in cell viability was observed at TMZ concentration of 160 μM. The lack of G2/M arrest in the TMZR cell lines is evident of the successful adaptation to TMZ (Fig. 1c, d, Additional file 2).

To demonstrate the clinical relevance of this study to GBM treatment, four patient-derived cell lines from the Human Glioblastoma Cell Culture (HGCC) biobank were selected for this study. These cell lines derived from surgical samples of GBM patients were maintained under conditions to preserve the original characteristics [49]. U3054MG and U3020MG but not U3008MG and U3117MG were resistant to TMZ (Fig. 1e). Particularly, U3054MG was derived from a recurrent GBM patient.

**Fig. 2:**
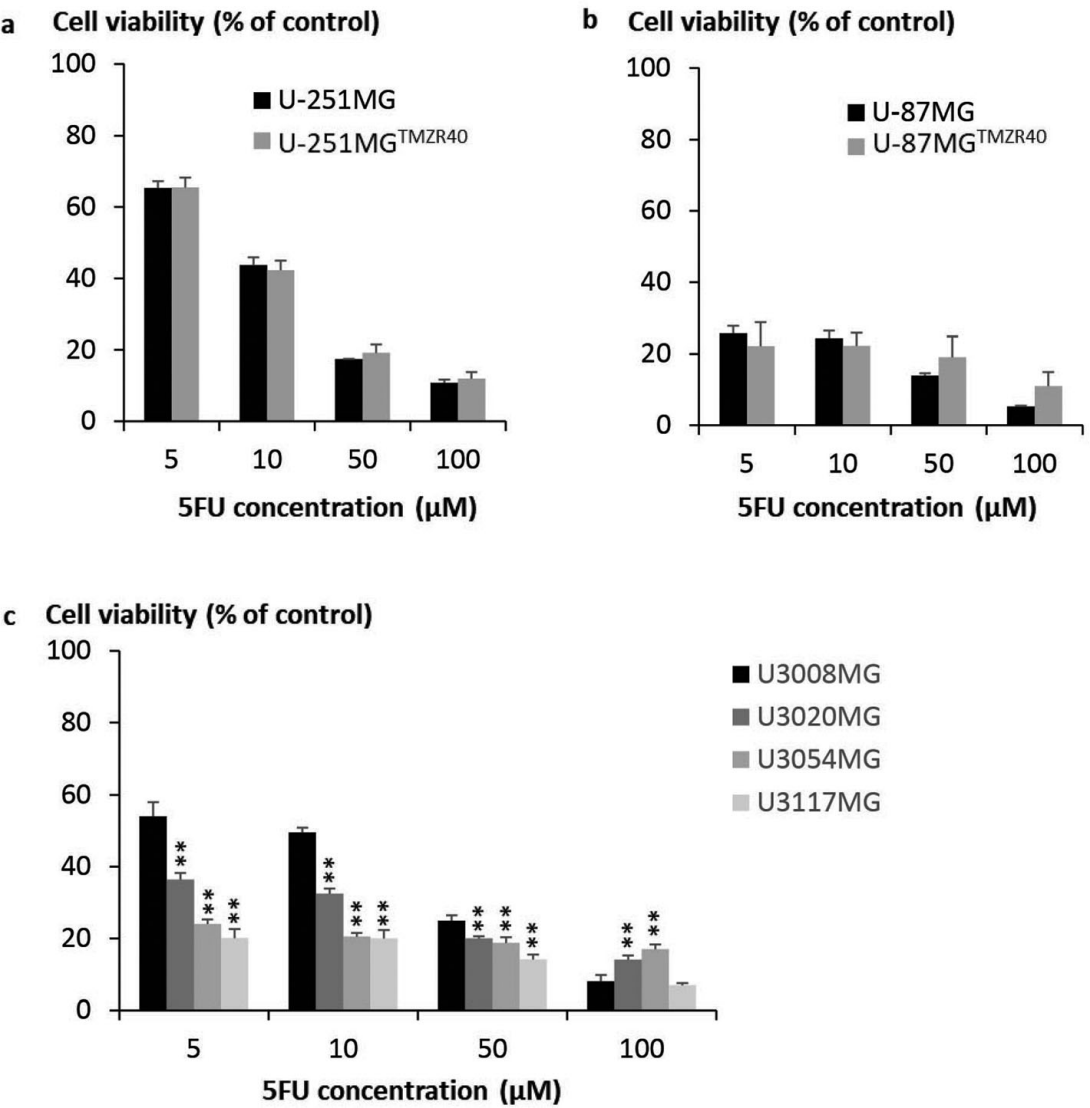
5FU sensitivity of various GBM cell lines. Quadruplicates of **a** U-251MG, **b** U-87MG and **c** patient-derived GBM cell lines (2500 cells) were plated. One day later, culture media were replaced with DMEM supplemented with 2% FBS and 5FU (0–100 μg/mL). Cell viability was determined using MTS assay 5 days later. The percentage of cell viability was calculated with no treatment controls set at 100%. Significant differences in 5FU sensitivities between U3008MG/U-251MG/U-87MG and other cell lines were calculated using two-tailed Student’s *t* test. **P < 0.005

Regardless of TMZ susceptibility, the glioma cell lines were sensitive to 5FU treatment, with the IC50 values ranging from 1 to 10 μg/mL (Fig. 2). U-251MG, U-251MG™^ZR40^ and U3008MG were the least sensitive towards 5-FU treatment, with IC50 of ~ 9 μg/mL. U3117MG is the most sensitive, with IC50 of 0.71 μg/mL. It is worthy to note that comparable 5FU response was also observed in both parental cell lines and their respective TMZ-resistant variants (Fig. 2a, b), indicative that the mode of actions of 5FU and TMZ may be distinct.

In line with this suggestion is that the expression of genes known to be involved in 5FU resistance (dihydropyrimidine dehydrogenase (DPYD), thymidylate synthase (TYMS), uridine monophosphate synthetase (UMPS), and ABCC5 transporter [51–54]) were largely unaltered on the development of TMZ resistance, with the exception of a small fold changes in the expressions of TYMS and UMPS in U-251MG™^ZR40^ (Additional file 3). There was also no distinct pattern in the *z*-score of the genes involved in 5FU resistance in the four patient-derived cell lines which were resistant to TMZ (Additional file 4).

### A scalable non-viral method for generation of therapeutically engineered MSCs

In order to be useful for downstream translational efforts, this non-viral method must be scalable in production. Previously, we improved transfection by the use of a low-speed centrifugation step which reduced cytotoxicity by minimizing the exposure of the cells to free polymers [43, 44]. However, the inclusion of such a centrifugation step predictably limits the scalability of the process, rendering scale-up production of transfected MSCs challenging.

To circumvent the need of a centrifugation step, we first evaluated the performance of five commercially available non-viral gene carriers in transfecting MSCs without the need for low-speed centrifugation (Additional file 5). The transfection efficiencies using GFP as a reporter showed a diverse range of performances. In line with other studies [43, 55, 56], increasing DNA and polymer amount did not improve transfection efficiency but reduced cell viability and the number of adherent cells. PEI outperformed the other gene carries where transfection with 500 ng pDNA complexed at the ratio of 1 μg pDNA to 3 μL PEI (1 mg/mL) resulted in 48% GFP+ cells and > 80% cell viability. Unless otherwise specified, the ratio of 1 μg pDNA to 3 μL PEI (1 mg/mL) was used in the following studies.

To further optimize transfection in AD-MSCs, Enhancer (Fusogenic lipid plus HDACi) was added to the culture during transfection to facilitate endosomal escape and microtubular trafficking of polyplexes intracellularly (Additional file 6) [43, 44]. In the presence of Enhancer, low pDNA amount of 300 ng was sufficient to achieve > 90% GFP+ cells. It is worth noting that scaling out and scaling up MSC modification with flat-bed (Additional file 7) and microcarriers in bioreactors (Additional file 8), respectively, are highly feasible. In both systems, the linearities of scaling out from 24-well plate to T175 flasks and scaling up from 1.9 to 47.5 cm^2^ of microcarriers were highly correlated to the number of AD-MSCs, with *R*^2^ close to 1. Hence, it is feasible that this transfection workflow can be extended to clinical scale production of therapeutically engineered MSCs.

### Phenotypic characteristics of AD-MSC remained unchanged post-transfection

**Fig. 3:**
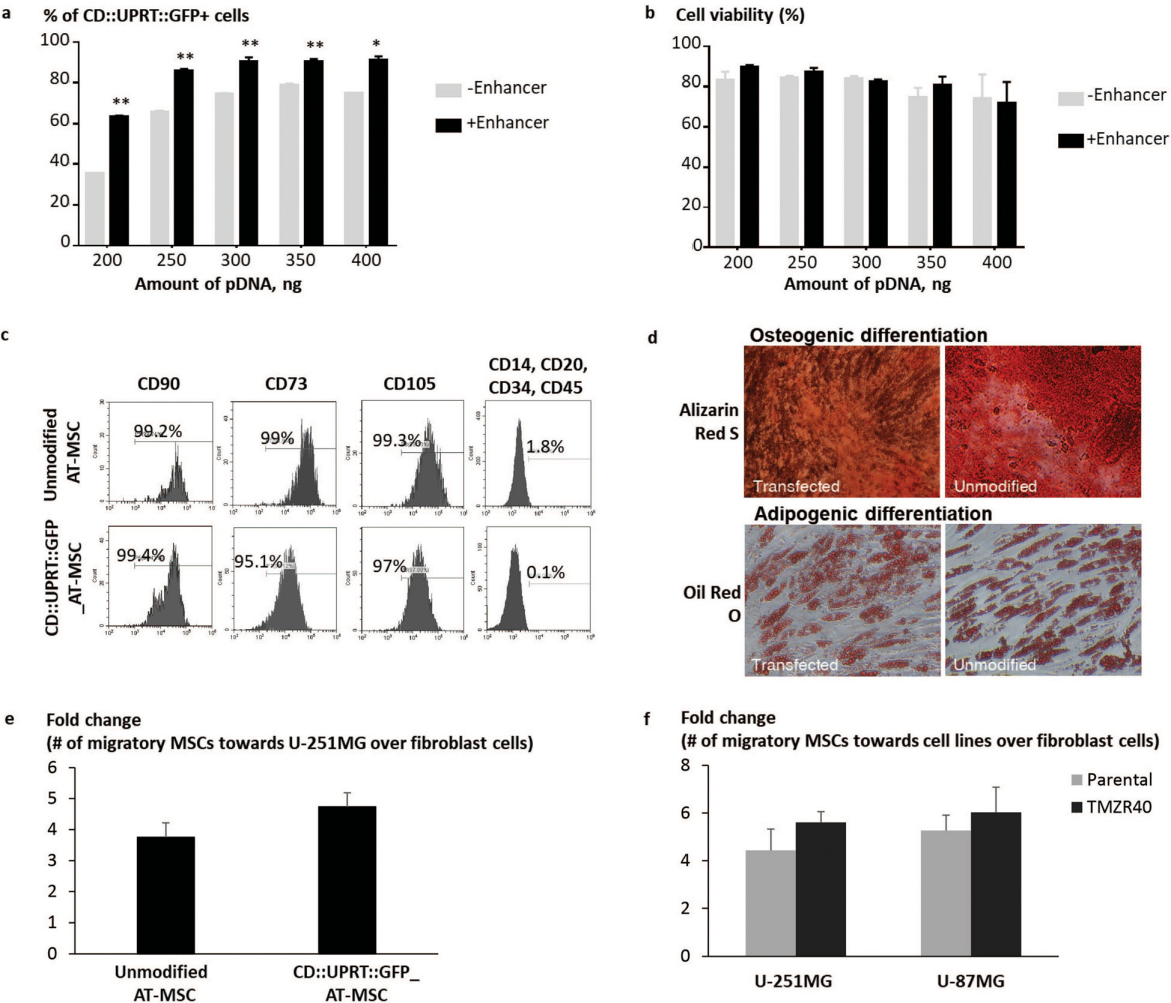
Generation of therapeutic AD-MSCs that fulfil the clinical requirement. Cells were transfected with LPEI at various pDNA amounts, ± Enhancer. Forty-eight hours later, cells were harvested for flow cytometry analysis to determine the percentage of CD::UPRT::GFP+ cells and **b** NC-3000 automated cell counting to measure cell viability with PI exclusion method. Graph bars present mean ± SD, *n* = 3. Significant differences ± Enhancer were calculated using two-tailed Student’s *t* test. **P < 0.005. AD-MSCs were transfected at 155 ng pDNA/cm^2^ in the presence of Enhancer. **c** AD-MSCs and CD::UPRT::GFP_AD-MSCs were labelled with fluorophore-conjugated antibodies and analysed by flow cytometry according to the manufacturer’s instructions. Isotype antibodies served as respective controls for gating. Histograms demonstrate the immunophenotypic profiles. **d** AD-MSCs and CD::UPRT::GFP_AD-MSCs were cultured in osteogenic or adipogenic differentiation medium for 14 or 21 days, respectively. The presence of calcium deposits stained with Alizarin red S indicates osteogenic differentiation of AD-MSCs. Oil Red O stained for oil droplets visible in the cells and was indicative of adipogenic differentiation. All images were captured at × 20 magnification. Migratory property of MSCs was evaluated using transwell assay. **e** Target cells (glioma cell lines or fibroblast) were plated in complete media. One day later, media were replaced with serum-free DMEM. CD::UPRT::GFP_AD-MSCs (transfected 1 day before the experiment) and un-transfected AD-MSCs were loaded onto the cell inserts. The inserts were transferred to the target cell cultures, respectively. Twenty-four hours later, cell migration was evaluated under a microscope by taking fluorescent images of cells stained with Hoechst 33342. **f** A similar study was performed to compare migration of modified MSCs towards parental and TMZ glioma cells. The fold change of AD-MSCs migrated towards cancer cells over fibroblast was calculated. Graph presents the mean of fold change + SD (*n* = 3)

Next, we transfected AD-MSCs with the therapeutic gene CD::UPRT::GFP and evaluated the characteristics of modified MSCs according to the clinical release criteria for cancer treatment (EU Clinical Trials Register number: 2012-003741-15) [57]. Transfection of AD-MSCs with PEI at 155 ng/cm^2^ in the presence of Enhancer resulted in > 75% of cells positive for the transgene as analysed by flow cytometry (Fig. 3a, Additional file 9) with > 80% viability of cells (Fig. 3b). The CD::UPRT::GFP_AD-MSCs retained the phenotype of MSCs as they showed > 90% expression of immunophenotypic markers (CD90, CD73 and CD105) with lineage negative population of less than 10% (CD14, CD20, CD34 and CD45) (Fig. 3c). The CD::UPRT::GFP_AD-MSCs were capable of differentiating into osteogenic and adipogenic lineages according to ISCT criteria (Fig. 3d, Additional file 10) [58]. Furthermore, for AD-MSCs to serve as cancer targeting vehicles, the processes used to modify them must not change their capacity for migration and homing [16, 59]. Despite the high transfection efficiency, the tumour tropism of the CD::UPRT::GFP_AD-MSCs was comparable to the unmodified MSCs in the presence of cancer cells in vitro (Fig. 3e). Primary fibroblast cells were used as a control to confirm the preferential migration of AD-MSCs towards cancerous cells. Comparable numbers of CD::UPRT::GFP expressing AD-MSCs (CD::UPRT::GFP_AD-MSCs) were found to migrate towards the parental and TMZR glioma cell lines (Fig. 3f), warranting the use of the strategy for targeting both TMZ-sensitive and TMZ-resistant cancer cells.

Depending on the route of administration, it may require up to 4 days for MSCs to nest around the tumour foci [33]. In a recent report, prodrug was administered 48–72 h after modified MSC administration to allow sufficient time for nesting of MSCs into the tumour vicinity [45]. We next examined the duration of the transgene expression. Here, cells were harvested 1 day post-transfection (D0) and seeded on cell culture vessel to monitor the expression of CD::UPRT::GFP over time. Notably, the high expression of CD::UPRT::GFP was retained over a period of 8 days post-transfection when Enhancer was used during PEI-mediated transfection (Additional file 11).

### In vitro evaluation of anticancer efficiency of CD::UPRT::GFP_AD-MSCs/5FC

We first established a coculture model of CD::UPRT::GFP_AD-MSCs and U-251MG to determine the effects of various concentrations of 5FC and durations of treatments on cell viability (Additional file 12). It is worthy to note that 100 μg/mL of 5FC did not affect the proliferation of unmodified MSC (Additional file 13). Next, we compared the potencies of AD-MSCs produced by transfection with Lipofectamine 3000 and PEI with or without the presence of Enhancer (Additional file 14). Evidently, significantly lower cell viabilities were observed with CD::UPRT::GFP_AD-MSCs modified by PEI plus Enhancers at all coculture ratios in comparison to AD-MSCs transfected with other protocols. The results suggested that the potency of MSCs driven CDEPT was highly dependent on the efficiency of the transfection methods (Additional file 11).

In addition, we compared the PEI/enhancer method with Lentivirus carrying the same transgene construct (Additional file 15a, c–e). AD-MSCs stably expressing CD::UPRT::GFP (AD-MSCs^CD::UPRT::GFP^) were established by lentivirus transduction, followed by selection with antibiotics [60, 61]. The extent of the number of cells modified by either method was comparable (Additional file 15b). Remarkably, CD::UPRT::GFP_AD-MSCs exhibited significantly higher anticancer potency than the viral modified MSCs (AD-MSCs^CD::UPRT::GFP^) at all coculture conditions. With 1% of CD::UPRT::GFP_AD-MSCs, ~ 70% reduction in cell number was observed, which was 50% higher than AD-MSCs^CD::UPRT::GFP^ (Additional file 15f). These data further support the use of non-viral method as a potential alternative to the lentiviral method in producing therapeutically engineered MSCs.

**Fig. 4:**
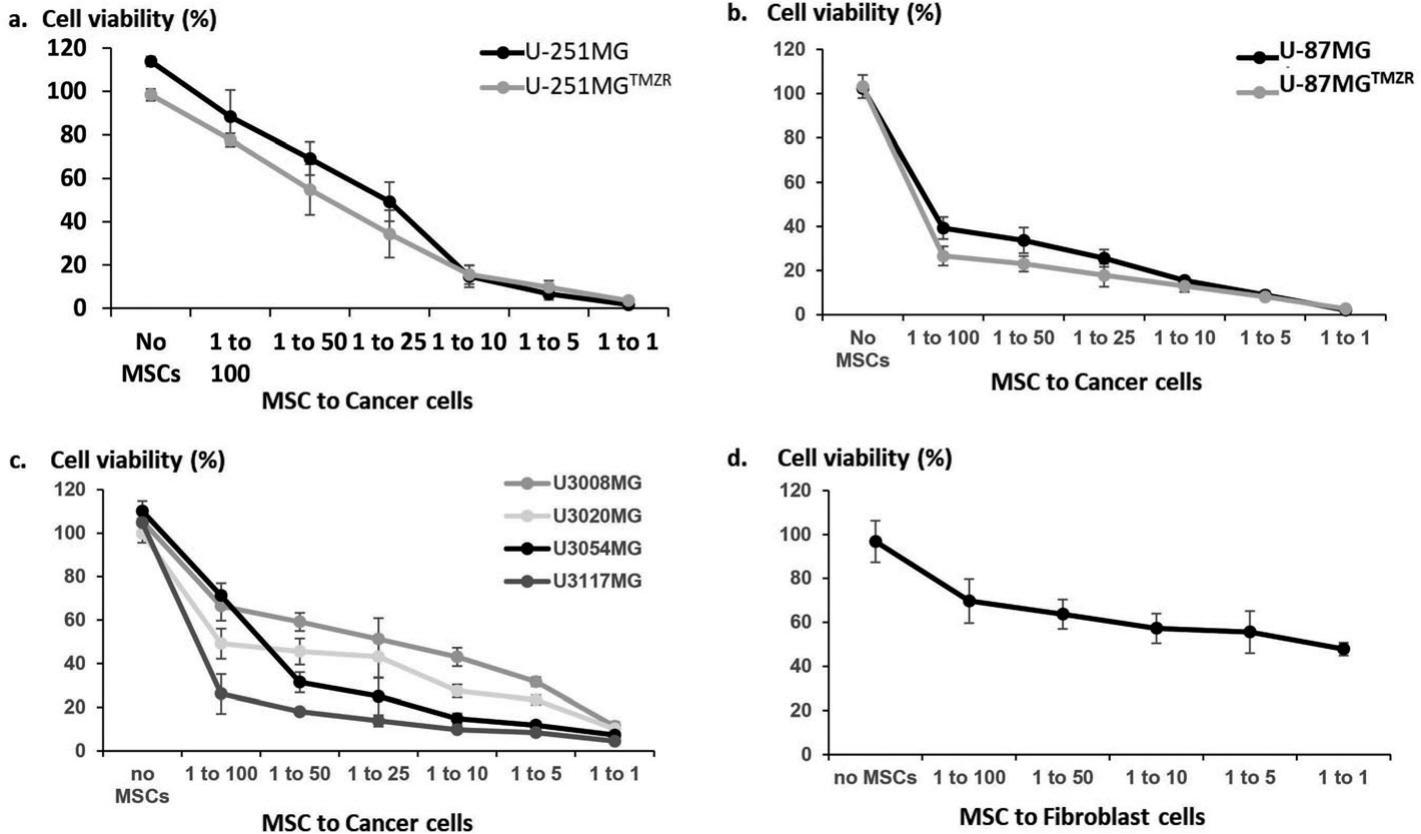
Anticancer effect mediated by CD::UPRT::GFP_AD-MSCs/5FC. Therapeutic AD-MSCs were cocultured with **a** U-251MG, U-251MG™^ZR40^, **b** U-87MG, U-87MG™^ZR40^, **c** U3008MG, U3020MG, U3054MG, U3117MG and **d** primary fibroblast cells. The cells were plated in 96-well plate in DMEM supplemented with 2% FBS. The therapeutic cells and target cells were mixed at ratios of 1 CD::UPRT::GFP_AD-MSC to 1, 5, 25, 10, 50 and 100 target cells. One day later, the culture medium was replaced with DMEM supplemented with 2% FBS in the presence or absence of 100 μg/mL 5FC. Seven days later, cell viability in the treatment conditions was evaluated spectrophotometrically by standard MTS assay. Cell viability (%) was defined as sample/control × 100%. Conditions without 5FC treatment served as controls. Conditions of no MSCs provide evidence that 5FC is non-toxic to the target cells. Data of biological quadruplicates presents as mean + SD

The therapeutic potential of CD::UPRT::GFP_AD-MSC in the presence of 5FC was further evaluated by the coculture of six glioma cell lines at various coculture ratios (Fig. 4, Additional file 16). The potencies of CD::UPRT::GFP_AD-MSCs/5FC in targeting various GBM cells were similar to the treatment with 5FU (Fig. 2), where parental and TMZR cells responded similarly (Fig. 4a, b). U3054MG and U3117MG showed comparable sensitivity to U-87MG where as little as 1% therapeutic cells was sufficient to reduce at least 50% of the population. It is noteworthy that U3054MG is derived from a recurrent GBM patient and has been shown to preserve the original characteristics of the primary tumour [49], further supporting the use of CD::UPRT::GFP_AD-MSCs/5FC for TMZR GMB treatment. On the other hand, U-251MG, U3008MG and U3020MG share similar response to the treatment where 4% of CD::UPRT::GFP_AD-MSC in the coculture was sufficient to reduce 50% of total cell viability (Fig. 4a, c). In contrast, a decrease of 50% of normal fibroblast cell coculture population only occurred with a ratio of 1 modified MSC to 1 fibroblast cell (Fig. 4d). This is indicative of a preferential sensitivity of GBM cells to the modified AD-MSCs in the presence of 5FC.

### In vivo evaluation of anticancer efficiency of CD::UPRT::GFP_AD-MSCs/5FC

**Fig. 5:**
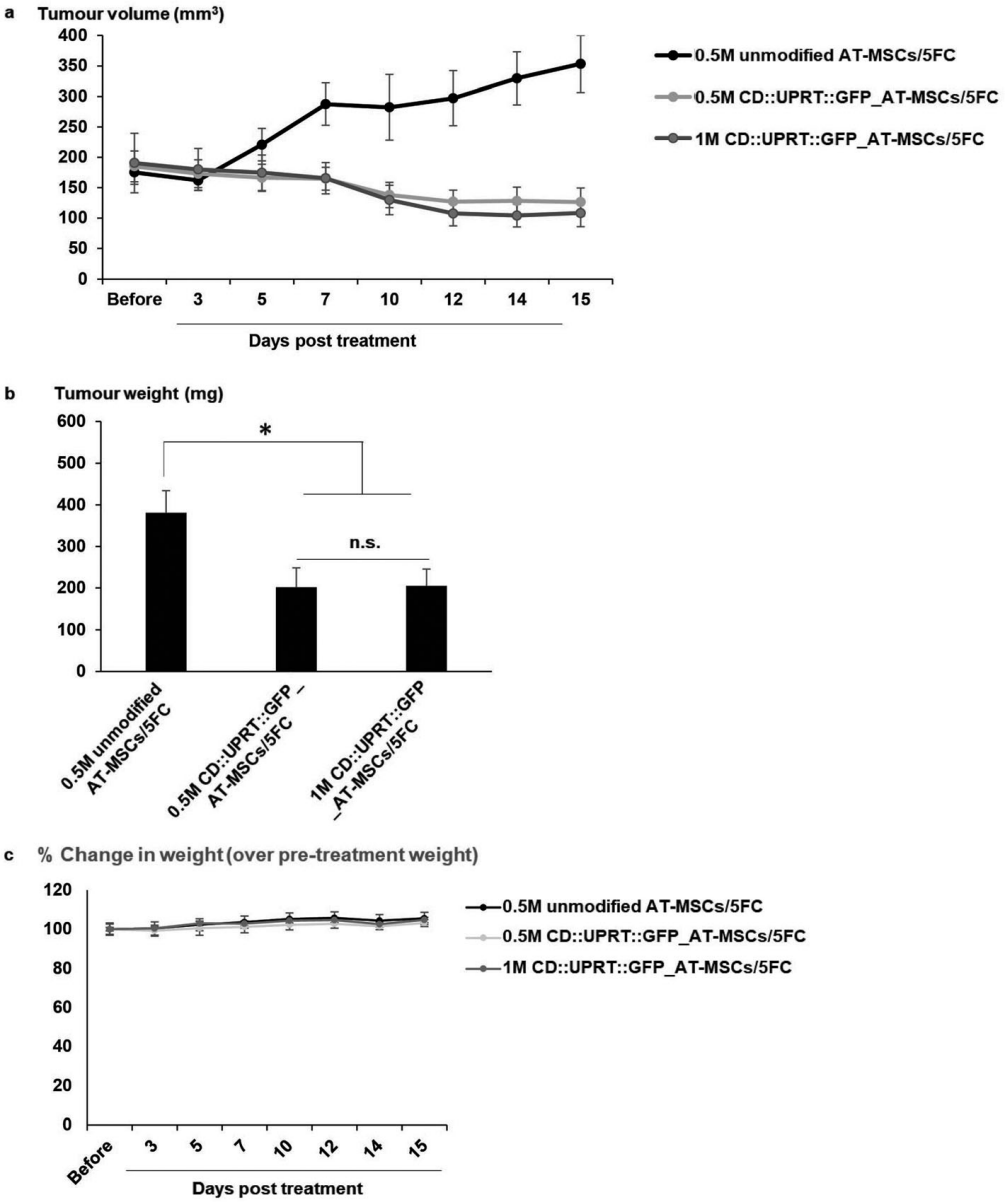
In vivo antitumoural effect of CD::UPRT::GFP_AD-MSCs in the presence of 5FC. To establish s.c tumour, 5 × 10^6^ U-251MG™^ZR40^cells were injected subcutaneously in dorsal flank regions. When tumour reached the target size, CD::UPRT::GFP_AD-MSCs or AD-MSCs were injected directly to the s.c. tumour. One day later, 500 mg/kg/day of 5FC was administered intraperitoneally for 4 consecutive days. The size of s.c tumour was measured with a vernier calliper up to 15 days post-AD-MSC administration. The group that received unmodified AD-MSCs/5FC serves as the control group. Tumour volume (mm^3^) was calculated according to the standard formula of *V* = (*W* × *W* × *L*)/2. **a** The line graph displays the tumour size in the three groups over time measured from *n* = 6 from each group. Data is presented as mean + SD, *n* = 6. **b** At the end of the study, the tumours were extracted for weight measurement. Graph bar demonstrates the average of tumour weight for each group. Data were expressed as mean + SD, *n* = 6. Significant differences between the three groups were calculated using two-tailed Student’s *t* test. *P < 0.05, n.s indicates statistical insignificance between groups. **c** The weight of the mice was measured at each time point prior to measurement of the tumour volume. The percentage of change in weight was defined as weight/weight before AD-MSC administration × 100%. The line graph displays average of the percentage in weight change + SD of 6 mice in each group

Extending the study, we next injected CD::UPRT::GFP_AD-MSCs directly into a subcutaneous (s.c) tumour model. CrTac:NCr-Foxn1nu female mice bearing subcutaneous human U-251MG™^ZR40^ tumours were divided into three groups, where one group served as a control using unmodified AD-MSCs and the other 2 groups with escalating dose of CD::UPRT::GFP_AD-MSCs. One day after the cells were injected, 5FC was administered daily for the next 4 consecutive days. Significant suppression of tumour growth was observed in the treatment groups but not the control group (Fig. 5a). With one administration of 500,000 CD::UPRT::GFP_AD-MSCs, the tumour size in the treatment group was at an average of 64% smaller than the control group, 15 days after the last 5FC administration. No further reduction of tumour size was observed with higher dose of therapeutic cells (Fig. 5b). All mice were observed every 2 days post-treatment and scored for any debilitating signs secondary to tumour growth or treatment, including hunched posture, obvious illness, laboured breathing, weight loss and inability to remain upright. Throughout the experiment, none of these adverse signs was observed. The weight of mice of all three groups was highly comparable with no significant weight loss over time (Fig. 5c).

**Fig. 6:**
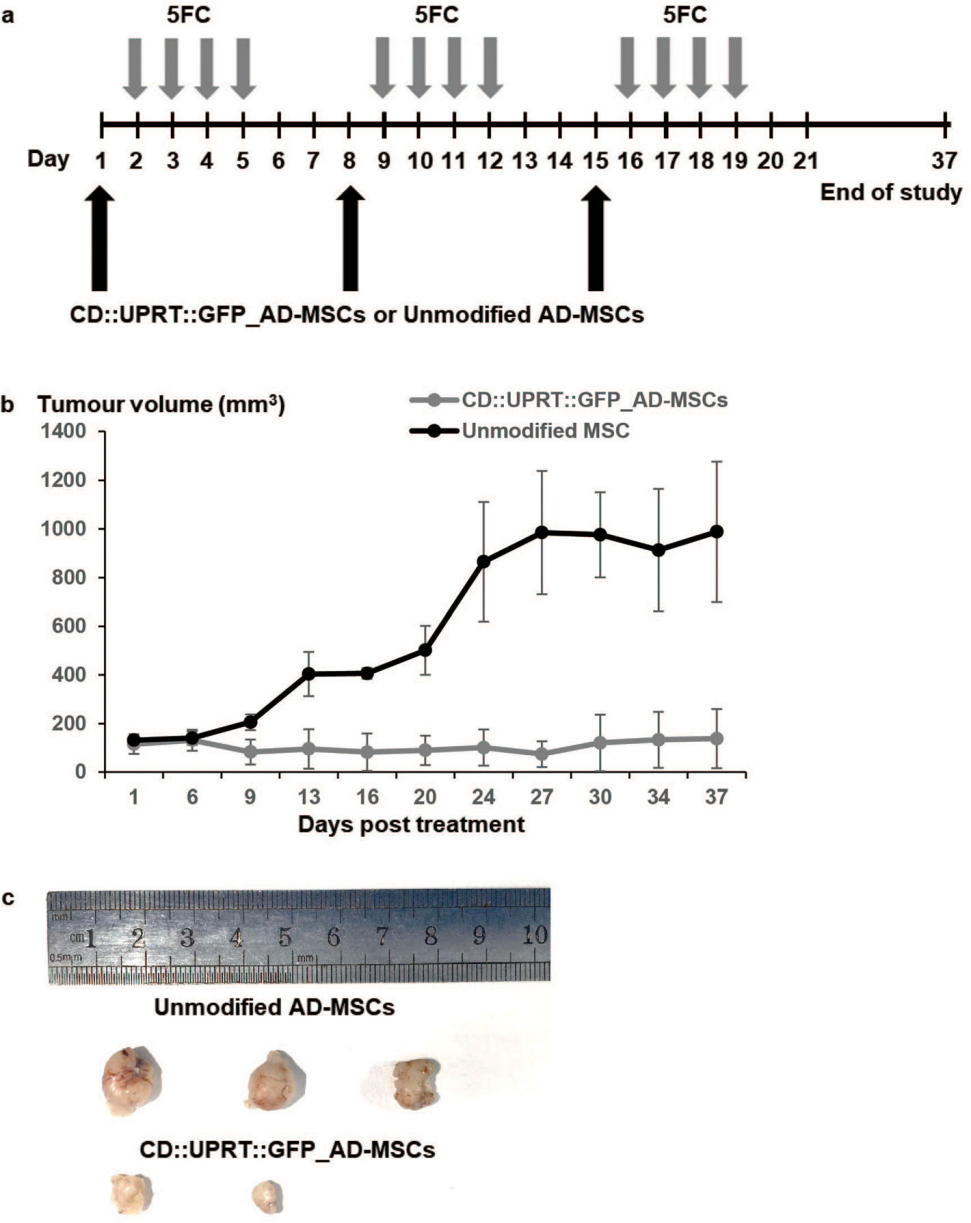
Tumour suppression up to 36 days with repeated dosage of CD::UPRT::GFP_AD-MSCs/5FC. **a** When the tumour size reaches the ~ 100 mm^3^, 1 × 10^6^ CD::UPRT::GFP_AD-MSCs or unmodified AD-MSCs were injected in the s.c tumour. All animals were treated with a daily dose of 500 mg/kg/day 5FC for 4 consecutive days starting 1 day post-MSC administration. The same cycle was repeated twice. **b** The tumour volume was measured every 3–5 days. Tumour volume (mm^3^) was calculated according to the standard formula of *V* = (*W* × *W* × *L*)/2. The line graph presents the average tumour volume + SD (*n* = 3). At the end of the experiment, mice were euthanized. **c** The tumours were extracted and fixed with 4% PFA. Images of tumours from each group are shown

Several reports have demonstrated an improvement of the therapeutic outcome of stem cell-driven prodrug therapy by repeated administration of therapeutic cells and 5FC [30, 62, 63]. To test this approach in vivo, cycles of 1 × 10^6^ CD::UPRT::GFP_AD-MSCs or unmodified AD-MSCs were injected into the subcutaneous tumour of U-251MG™^ZR40^ and repeated administration of 5FC every week as illustrated in Fig. 6a. The control group was treated with unmodified AD-MSCs and exposed to 5FC similarly. The results showed that this treatment regime could suppress tumour growth of up to 36 days where the tumour burden was reduced by more than 85% by the end of the study (Fig. 6b). Notably, tumour bulk was not detectable in one of the mice in the treatment group (Fig. 6c), suggesting the potential complete suppression of tumour growth.

## Discussion

MSC-driven prodrug therapy has gained recent attention as promising treatment of solid tumours, including GBM [13, 28–30]. Currently, the application of unmodified MSC in cancer is not under clinical investigation as MSCs have shown pro- or antitumour property, depending on the donor and types of MSC [64]. Engineering of MSCs with therapeutic genes is necessary as it offers a safer and more efficient cancer therapy as compared to the unstable and heterogenous unmodified cells [15, 65, 66]. The significant challenge here is then to efficiently deliver the desired therapeutic gene into MSCs so as to generate cells with high payload, high viability and yet with no changes in phenotypes, ideally, without the use of virus. To this end, we have demonstrated the feasibility of a highly scalable transfection method that outperformed viral gene delivery system in engineering AD-MSCs for GBM therapy and potentially other cancer indications.

To date, non-viral methods are not commonly used in the modification process of cell and gene therapy simply because of poor transfection [35, 38, 39]. As one of the release criteria in clinical trials requires > 75% cells expressing the therapeutic product [18, 45], low transfection efficiency is unacceptable and will require further enrichment of the transgene expressing cell population. While enrichment of the AD-MSC post-transfection is possible through antibiotic selection, this can be a challenge as replicative senescence of MSCs will greatly limit production scalability [67]. In the presence of transfection Enhancer, it is now possible to achieve > 90% modification efficiency, overcoming the challenges of using non-viral system in generating therapeutic MSCs.

In addition to high transfection efficiency, we showed that this method outperformed viral vector by producing MSCs with substantially higher gene expression, where the higher payload should result in greater potency in cancer killing. Consistent with this suggestion is that AD-MSCs transfected with PEI/Enhancer exhibited significantly higher potency in cancer killing than the virally modified AD-MSCs. With as little as 1% therapeutic cells, CD::UPRT::GFP_AD-MSCs killed 50% more cells. This is due to the efficient conversion of 5FC by the high payload of therapeutic gene as suggested by the comparable potency of CD::UPRT::GFP_AD-MSCs/5FC and 5FU. At 100 μg/mL of 5FC, CD::UPRT::GFP_AD-MSC plated at a 1:5 ratio with cancer cells was as potent as 100 μg/mL of 5-FU (Fig. 2a).

The use of 5FU in GBM treatment is hampered by the dose-limiting toxicities before achieving sufficient antitumour efficacy [68]. Hence, the MSC-driven prodrug therapy using CD locally converting the non-toxic prodrug 5-FC into 5-FU may circumvent such limitations. MSCs have the ability to nest to the glioma stem cells that reside at the vascular niche [69]. As shown in previous pre-clinical studies, viral transduced CD expressing MSCs are found localized to tumour regardless of sites of administration into either the striatum [30], intracranial site [29], brain tumour [28] or the brain tissue surrounding the postoperative resection cavity [13], suggesting the potential of MSCs to serve as a GBM targeting vehicles. Unlike a previous report of the reduced migratory property of MSCs when overexpressing a transgene [42], the highly overexpressed CD::UPRT::GFP cells did not show a significant change in the tumour tropism. This property is particularly relevant to the use of gene-modified MSCs for targeting highly infiltrative GBM and may serve as an adjuvant treatment to the standard of care to improve the clinical outcome of GBM patients.

Currently, MSCs are mostly cultured on planar culture platforms, such as tissue culture plates and T-flasks [70, 71]. As a proof-of-concept, we were able to scale the production of modified cells on planar surfaces linearly. This culture setup is relatively easy to implement where larger cell quantities can be attained by scaling out with an increasing number of plates with larger surface areas. We have successfully transfected 1.5 × 10^8^ MSCs at > 80% efficiency in multiple one-layer CellSTACK vessels for an ongoing investigational study in large animals (data not shown). Doubling the production to 2.5 × 10^8^ cells in a 10-chamber vessel should be feasible, similar to that previously demonstrated in the scale-out of MSC expansion to 50–70 of such vessels [72]. While the scale-out approach is able to generate clinically relevant intracranial dose of 10–50 million cells for GBM treatment [18], it can become impracticable in terms of manual labour and incubator space requirements. For these reasons, automated cell factory manipulator (up to 40-layer vessel) and microcarrier-based bioreactor system (scale-up approach) are preferred when producing very large amounts of cells [73, 74]. In view of the foreseeable need, we have shown the efficient transfection of cells on microcarriers and the next step is to scale-up by design transferring the process to stirred tank bioreactor cultures [72].

## Conclusion

Given the better therapeutic efficacy of non-virally modified, this study showed that AD-MSCs can be engineered to overexpress a therapeutic transgene and that these cells outperformed viral modified cells. To further the use of these therapeutic AD-MSCs for targeting tumour in vivo, studies are underway to demonstrate safety and efficacy in pre-clinical orthotopic GBM models using patient-derived cells [75]. In conclusion, non-viral gene modification of AD-MSCs can serve as an effective cellular vehicle for prodrug therapy of solid tumours including GBM.

## Supplementary information

**Additional file 1.** Schematic diagram of the CD::UPRT::GFP plasmid construct.

**Additional file 2.** Flow cytometry profiles of the cell cycle of the TMZ sensitive and resistant cell lines in the presence and absence of TMZ treatment. The number of cells acquired during flow cytometry measurement is 5000 per sample. The analysis of the cell cycle is presented in Fig. 1c and d.

**Additional file 3. **Gene expression of genes related in 5FU resistance pathway. Fold change of expression for DPYD, TYMS and UMPS in **a** U-251MG and **b** U-87MG TMRZ cell lines were calculated in relative to their respective parental cell lines. Similarly, changes in the expression of ABCC5 transporter were calculated accordingly for **c** U-251MG and **d** U-87MG TMZR cell lines. All samples were analysed in triplicates. The fold change in the expressions of the gene of interest was calculated after normalization to the house keeping gene GAPDH. Line graph shows the average fold change in gene expression, mean + SD (*n* = 3). Significant differences between parental and TMZR cells were calculated using unpaired, two-tailed Student’s t-test. *p*-value < 0.05 is represented by *, p-value < 0.005 is represented by **.

**Additional file 4.** Gene expression z-score of patient derived cell lines for 5-FU pathway. DPYD, TYMS, UMPS, TYMP and ABCC5 expression levels are shown. The information was obtained from www.hgcc.se Dated: 3 Jun 2019.

**Additional file 5. **Determination of the transfection efficiencies of various commercial carriers in AD-MSCs. One day post seeding of 50,000 AD-MSCs, cells were transfected with different gene carriers at various GFP encoded plasmid (PF463-CMV-GFP, PlasmidFactory) and gene carrier amounts. AD-MSCs were transfected with **a** Lipofectamine 3000, **b** Polyfect, **c** Transficient, and **d** Turbofect according to the manufacturer’s instructions. **e** For cells transfected with PEI, the protocol is detailed in methods and materials section. Two-day post transfection, AD-MSCs were harvested. Transfection efficiency and cell viability (Propidium Iodide exclusion assay) were determined using NucleoCounter®NC-3000™. For each gene carrier, the left and right graph bar presents % of GFP+ cells and % of cell viability (% of PI- population), respectively. Data represents mean ± SD, *n* = 3.

**Additional file 6. **Supplementation of Enhancer to improve transfection in AD-MSCs. One day post seeding of 50,000 AD-MSCs, cells were transfected by 150-500 ng of PF463-CMV-GFP complexed with PEI at 1 μg pDNA to 3 μL PEI, in the presence or absence of Enhancer. **a** Two days post-transfection, the fluorescent images were captured at 4x magnification. Representative images are presented. **b** After which, cells were harvested for FACS analysis. Untransfected MSC served as negative control for gating. Data represents mean ± SD, n = 3 (Bar graph).

**Additional file 7. **Scaling out MSC modification process on flat-bed culture. AD-MSCs were seeded in 6-well plate, T25, T75 and T175 flasks at 20,000/cm2. One day post seeding, cells were transfected with 150 ng PF463-CMV-GFP/ cm2 complexed at 1 μg pDNA to 3 μL PEI. Two-day post transfection, cells were harvested for analysis. Total number of cells harvested from each culture vessels were determined by the automated cell counter NC-3000. After which, the % of GFP+ cells measured with flow cytometry. **a** The scatter plot represents absolute number of GFP+ cells collected from each culture vessel; deduced from the readout of cell counting and flow cytometry of the GFP expression. Data of biological triplicates are expressed as mean + SD. **b** Representative images present the flow cytometry profile of unmodified and modified AD-MSCs harvested from T175 flasks.

**Additional file 8. **Scaling up MSC modification process on microcarrier culture. AD-MSCs were seeded at 28,000/cm2 on Cytodex® 3 microcarriers (total surface area of 1.9 cm2) in 24-well non-adherent plates, with agitation speed of 50 rpm for 24 h before transfection. Similar to flat-bed transfection, the polymer and DNA complex (at 200-300 ng of pDNA) were added to the cell culture using a dropwise manner after 15 min incubation. Cells were transfected in the presence or absence of the enhancer. Two-day post transfection, **a** the fluorescent images were captured at 10x magnification. Representative images are shown. **b** After which, cells were harvested through trypsinization. To separate the cells from microcarrier, the suspension cells were filtered through 70 μm cell strainer. The transfection efficiencies were determined through flow cytometry analysis. Cell viability based on PI exclusion was determined with NC-3000 automated cell counter. The combination chart presents % of GFP+ cells (graph bar) and cell viability (mark type) Data of biological triplicates are expressed as mean + SD. **c** For microcarriers with total surface area of 1.9 cm2 and 9.5 cm2, cells were transfected in 24-well non-adherent plates. To further scale up the transfection process, AD-MSCs were seeded on Cytodex® 3 (total surface area of 47.5 cm2) at 28,000 cells/cm2 in 125 mL Erlenmeyer flasks. One day later, cells were transfected in the presence of Enhancer. The amount of DNA was fixed at 150 ng/cm2 of microcarriers. Two-day post transfection, transfection efficiency was analysed. The scatter plot represents absolute number of GFP+ cells collected from each culture vessel; deduced from the readout of cell counting and flow cytometry analysis of the GFP expression. Data are expressed as mean + SD, *n* = 3.

**Additional file 9.** Representative images and FACS profile of AD-MSCs transfected with 300 ng of CD::UPRT::GFP plasmid, in the presence or absence of the Enhancer.

**Additional file 10.** Overlay of adipogenic differentiated AD-MSCs with GFP fluorescent image provides direct evidence of adipogenic differentiation of CD::UPRT::GFP expressing AD-MSCs. The images were captured at 20x magnification. Then, a representative set of images were cropped and enlarged.

**Additional file 11. **Prolonged expression of CDy::UPRT::GFP. One day post-transfection (D0), cells were harvested and seeded in 24-well plate for further incubation. Cells were harvested for CD::URPT::GFP expression analysis after 1, 3, 5, 7 days (D1, D3, D5, D7) of incubation. For each time point, cells were fixed with 4% PFA for batch analysis. The % of GFP+ cells were determined by flow cytometry analysis. Graph presents mean + SD (n = 3). Significant differences between PEI + Enhancer and other protocols were calculated using two tailed Student’s t-test. **, *P* < 0.005.

**Additional file 12. **Coculture duration and 5FC concentration required for optimal in vitro cytotoxicity study. **a** CD::UPRT::GFP_AD-MSCs were cultured with U-251MG in low serum DMEM, in the presence or absence of 100 μg/mL 5FC. With pre-seeded U-251MG, the therapeutic cells were added at ratios of 1 CD::UPRT::GFP_AD-MSCs to 10, 5, 1 cancer cells. Three, five and seven days later (D3, D5, D7), cell viability in the treatment conditions was evaluated spectrophotometrically by MTS assay. Cell viability was defined as sample/control × 100%. Conditions without 5FC treatment served as controls for respective AD-MSCs to U-251MG ratios. Data of biological quadruplicates were expressed as mean + SD. **b** Similar study was performed to determine the 5FC concentration required for maximal cytotoxic effect. The coculture of CD::UPRT::GFP_AD-MSCs and U-251MG cells were treated with various concentration of 5FC for 7 days. At the end of the experiment, MTS assay was performed. Data of biological quadruplicates are expressed as mean + SD. Significant differences between 50 μg/mL of 5FC and other 5FC concentrations were calculated using two tailed Student’s t-test. *, *P* < 0.05.

**Additional file 13. **Unmodified AD-MSCs were seeded in 24-well plate at 2500/cm^2^. One day later, the culture media is replaced with low serum DMEM, in the presence or absence of 100 μg/mL 5FC. Cells were further incubated for 4 days. At the end of experiment, cells were trypsinised and subjected to cell count with the automated cell counter NC-3000. Graph bar presents the absolute cell number harvested from triplicates of each condition (mean + SD).

**Additional file 14. **AD-MSCs generated with PEI plus Enhancers outperformed other methods. AD-MSCs were transfected with CD::UPRT::GFP plasmid using various protocols. Twenty-four hours post-transfection, CD::UPRT::GFP_AD-MSCs were cocultured with U-251MG in DMEM supplemented with 2% FBS, in the presence or absence of 100 μg/mL 5FC. The therapeutic cells were mixed at ratios of 1 CD::UPRT::GFP_AD-MSC to 25, 10, 5, 1 cancer cells. Seven days later, cell viability in the treatment conditions was evaluated spectrophotometrically by MTS assay. Cell viability was defined as sample/control × 100%. Conditions without 5FC treatment served as controls. No MSC condition suggests lack of 5FC cytotoxicity in cancer cells. Data presents mean + SD of the biological quadruplicates. Statistical differences between Lipofectamine 3000 and other methods were calculated using two tailed Student’s t-test. **, *P* < 0.005.

**Additional file 15. **Non-viral modified AD-MSCs displayed higher anticancer potency. AD-MSCs were modified with PEI plus Enhancer and lentivirus carrying CD::UPRT::GFP at various pDNA amount and MOI, respectively. Two- or five-day post transfection or infection, **a** the fluorescent images were captured. Representative images are shown. **b** Then, cells were trypsinised, pelleted and resuspended in 1XPBS for flow cytometry analysis. Cell modification efficiency was calculated as % of CD::UPRT::GFP+ cells normalized to the total number of cells as quantified by FACS. **c** The mean RFU of AD-MSCs modified with lentivirus at MOI5 and non-viral method at 75 ng pDNA as measured by FACS is presented, *n* = 3. **d** The merged flow cytometry histogram displays the expression profile of the unmodified AD-MSCs (grey), AD-MSCs modified with 75 ng pDNA (Green) and lentivirus at MOI5 (orange). Population with FITC higher than 106 RFU is defined as population with high transgene expression. **e** The % of population with high transgene expression is presented with the bar graph, n = 3. **f** AD-MSCs modified transiently with PEI + Enhancer or stably with lentivirus were harvested for coculture with HT1080 cell line. One day later, cells were treated with 100 μg/mL of 5FC for 5 days. At the end of the experiment, MTS assay was performed to measure the cell viability. Bar graph represents mean of cell viability (%) ± SD, *n* = 4. Significant differences between PEI + Enhancer and Lentivirus were calculated using two tailed Student’s t-test. **, P < 0.005.

**Additional file 16.** CD::UPRT::GFP_AD-MSCs were cocultured with U251MG™^Z^ or U87MG™^Z^ at the ratio of 1:10, in the presence or absence of 5FC. After seven days of incubation, images of the culture were captured. Representative images are presented.

## Data Availability

Data are available within the article or its supplemental materials.

## Abbreviations

TMZR: Temozolomide-resistant
5FC: 5-Fluorocytosine
MSCs: Mesenchymal stem cells
CDEPT: Cell-directed enzyme prodrug therapy
GBM: Glioblastoma multiforme
CD::UPRT::GFP: Yeast cytosine deaminase::uracil phosphoribosyl-transferase::green fluorescent protein
5FU: 5-Fluorouracil
TMZ: Temozolomide
NSC: Neural stem cell
UPRT: Uracil phosphoribosyl-transferase
AD-MSCs: Human adipose-derived mesenchymal stem cells
DMEM: Dulbecco modified Eagle medium
FBS: Fetal bovine serum
PEI: Polyethylenimine MAX
CD::UPRT::GFP_AD-MSC: CD::UPRT::GFP expressing AD-MSC
AD-MSCs^CD::UPRT::GFP^: AD-MSCs stably expressing CD::UPRT::GFP
s.c: Subcutaneous
