# Supplementary figures and images for: A facile and scalable in production non-viral gene engineered mesenchymal stem cells for effective suppression of temozolomide-resistant (TMZR) glioblastoma growth
Source: Stem Cell Res Ther. 2020 Sep 11;11:391. doi: 10.1186/s13287-020-01899-x (PMC7488524; doi:10.1186/s13287-020-01899-x)

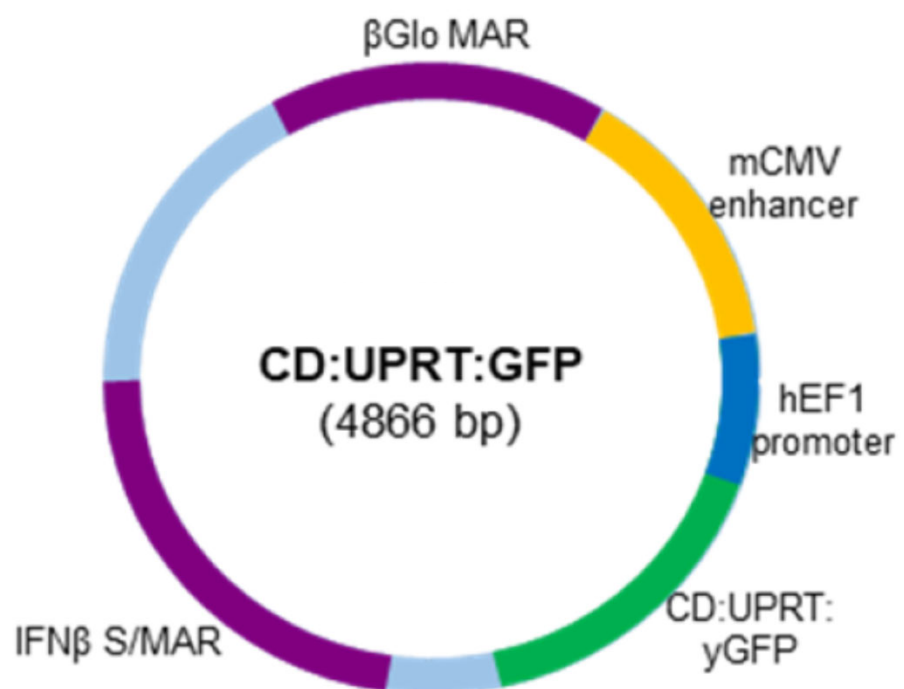

Supplement: Supplementary file 1 — Additional file 1. Schematic diagram of the CD::UPRT::GFP plasmid construct. [file 13287_2020_1899_MOESM1_ESM.pdf]

Control

U-87MG

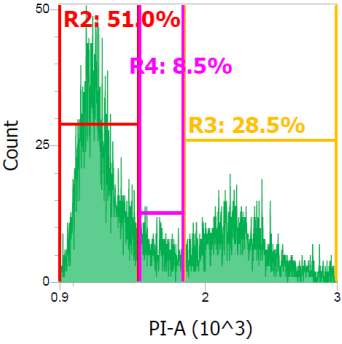

U-87MG<sup>TMZR40</sup>

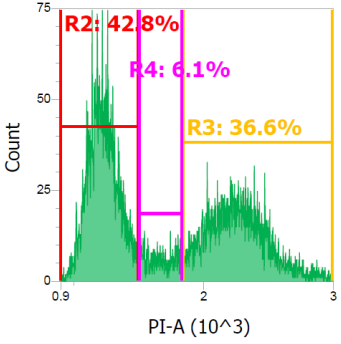

10  $\mu$ M  
TMZ

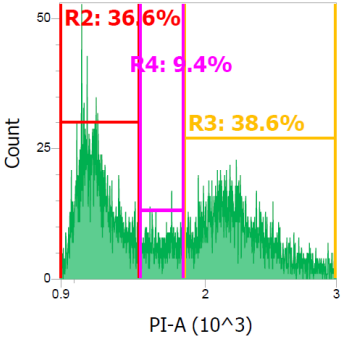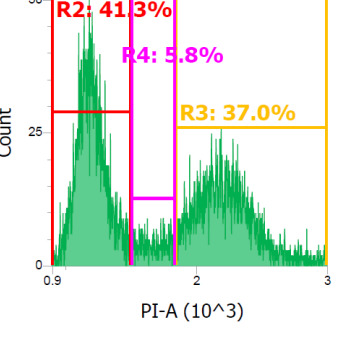

40  $\mu$ M  
TMZ

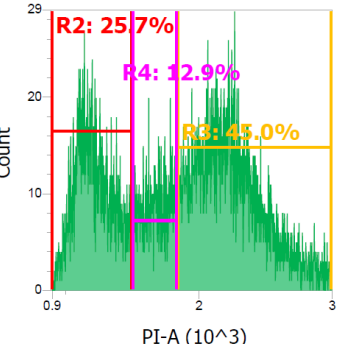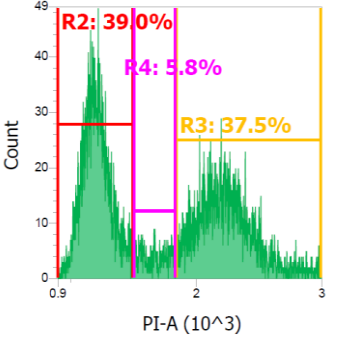

100  $\mu$ M  
TMZ

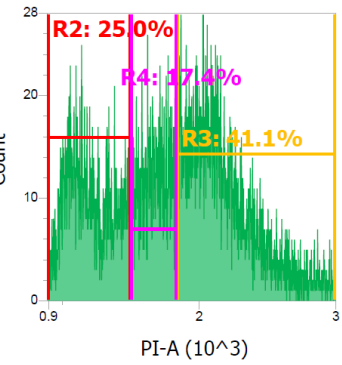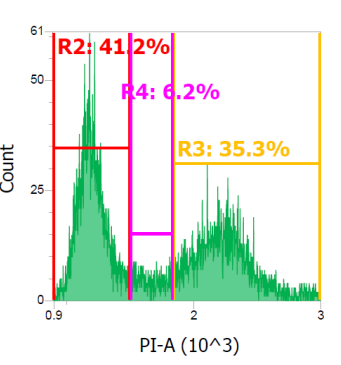

Supplement: Supplementary file 2 — Additional file 2. Flow cytometry profiles of the cell cycle of the TMZ sensitive and resistant cell lines in the presence and absence of TMZ treatment. The number of cells acquired during flow cytometry measurement is 5000 per sample. The analysis of the cell cycle is presented in Fig. 1c and d. [file 13287_2020_1899_MOESM2_ESM.pdf]

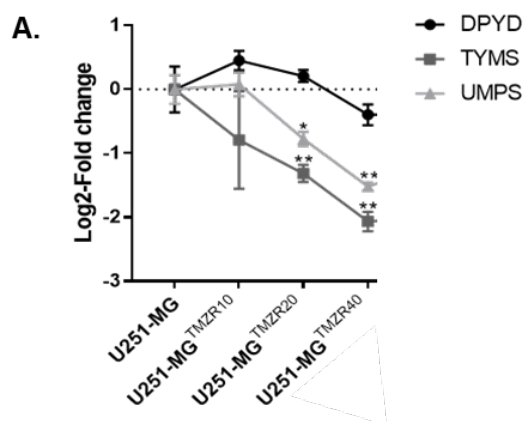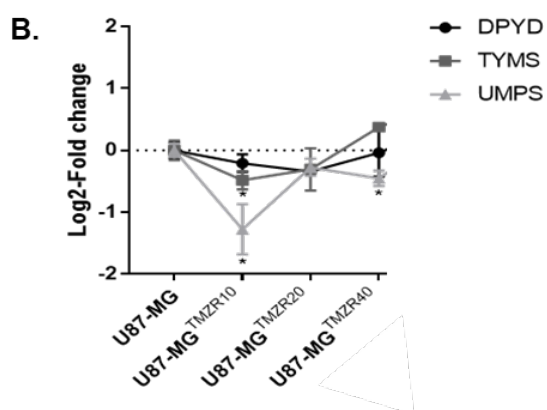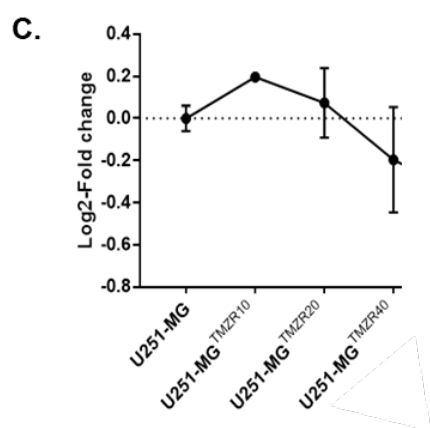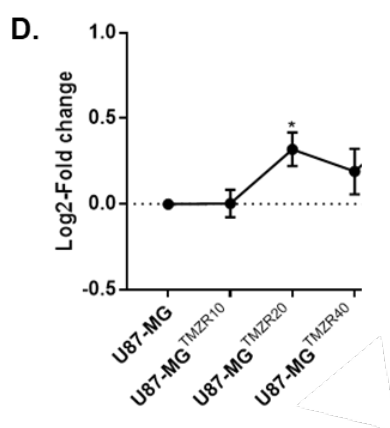

Supplement: Supplementary file 3 — Additional file 3. Gene expression of genes related in 5FU resistance pathway. Fold change of expression for DPYD, TYMS and UMPS in a U-251MG and b U-87MG TMRZ cell lines were calculated in relative to their respective parental cell lines. Similarly, changes in the expression of ABCC5 transporter were calculated accordingly for c U-251MG and d U-87MG TMZR cell lines. All samples were analysed in triplicates. The fold change in the expressions of the gene of interest was calculated after normalization to the house keeping gene GAPDH. Line graph shows the average fold change in gene expression, mean + SD (n = 3). Significant differences between parental and TMZR cells were calculated using unpaired, two-tailed Student’s t-test. p-value < 0.05 is represented by *, p-value < 0.005 is represented by **. [file 13287_2020_1899_MOESM3_ESM.pdf]

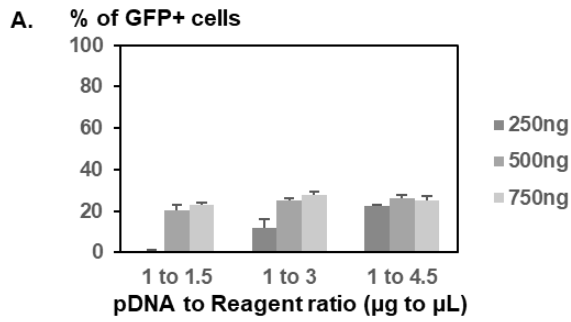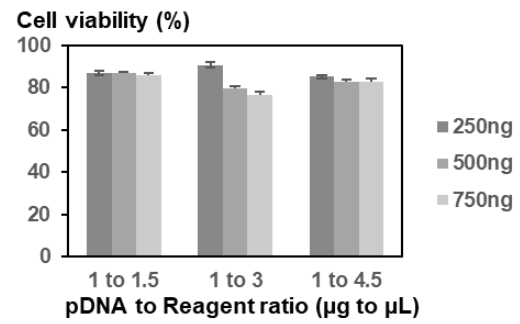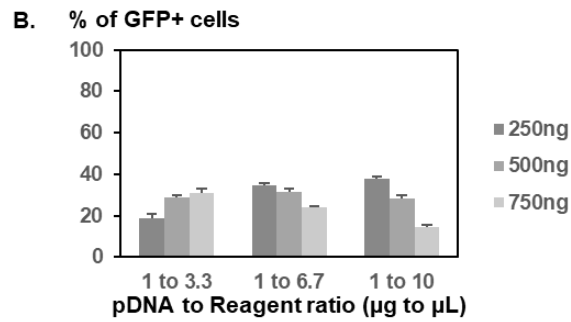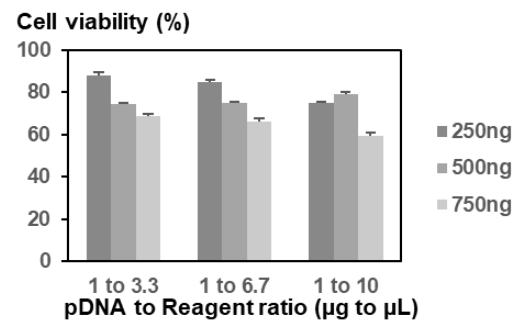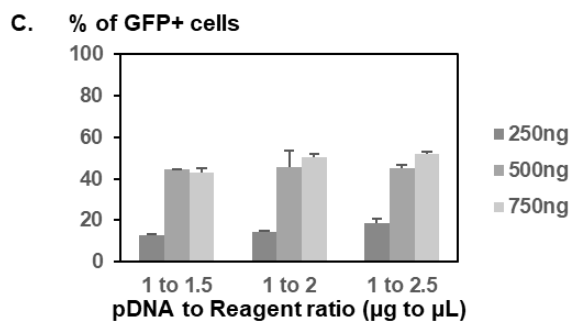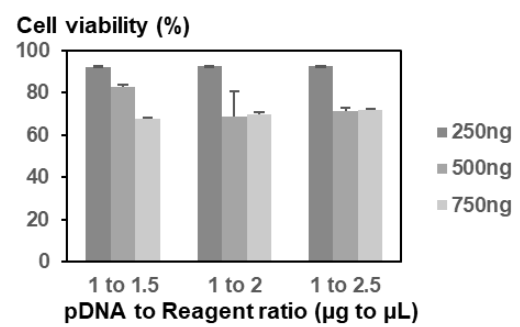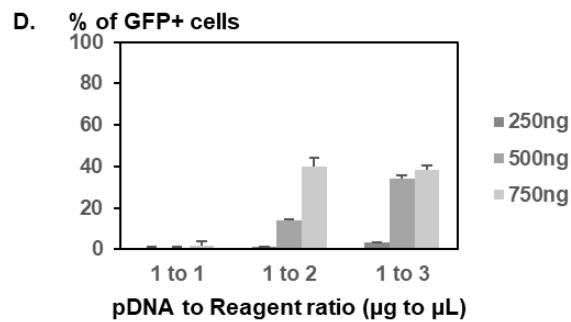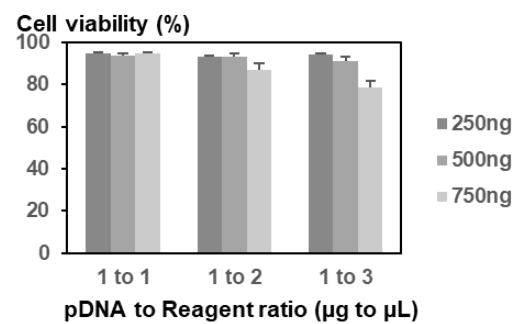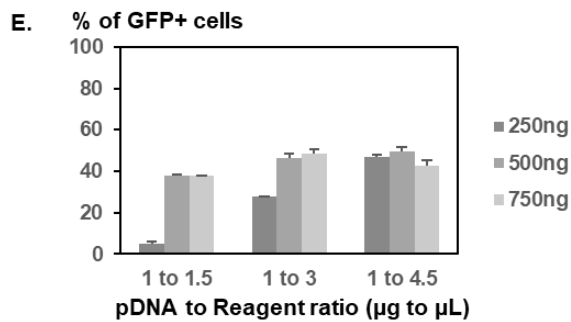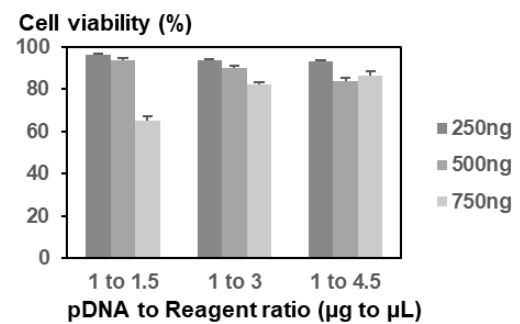

Supplement: Supplementary file 5 — Additional file 5. Determination of the transfection efficiencies of various commercial carriers in AD-MSCs. One day post seeding of 50,000 AD-MSCs, cells were transfected with different gene carriers at various GFP encoded plasmid (PF463-CMV-GFP, PlasmidFactory) and gene carrier amounts. AD-MSCs were transfected with a Lipofectamine 3000, b Polyfect, c Transficient, and d Turbofect according to the manufacturer’s instructions. e For cells transfected with PEI, the protocol is detailed in methods and materials section. Two-day post transfection, AD-MSCs were harvested. Transfection efficiency and cell viability (Propidium Iodide exclusion assay) were determined using NucleoCounter®NC-3000™. For each gene carrier, the left and right graph bar presents % of GFP+ cells and % of cell viability (% of PI- population), respectively. Data represents mean ± SD, n = 3. [file 13287_2020_1899_MOESM5_ESM.pdf]

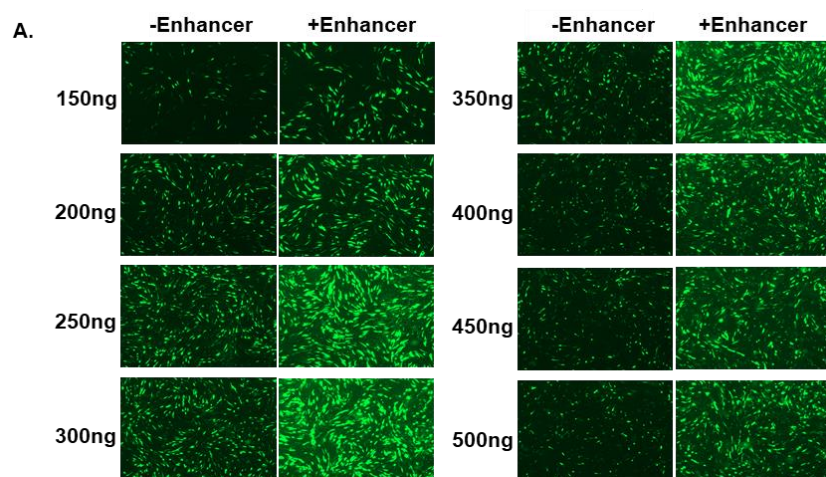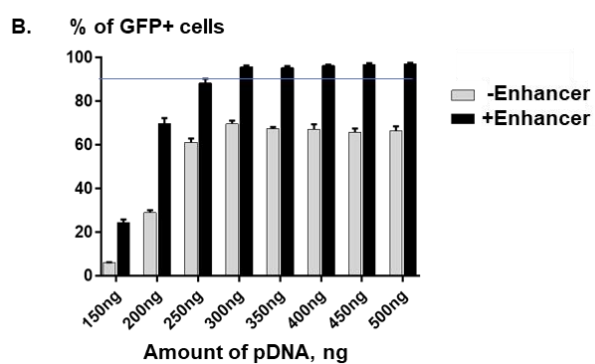

Supplement: Supplementary file 6 — Additional file 6. Supplementation of Enhancer to improve transfection in AD-MSCs. One day post seeding of 50,000 AD-MSCs, cells were transfected by 150-500 ng of PF463-CMV-GFP complexed with PEI at 1 μg pDNA to 3 μL PEI, in the presence or absence of Enhancer. a Two days post-transfection, the fluorescent images were captured at 4x magnification. Representative images are presented. b After which, cells were harvested for FACS analysis. Untransfected MSC served as negative control for gating. Data represents mean ± SD, n = 3 (Bar graph). [file 13287_2020_1899_MOESM6_ESM.pdf]

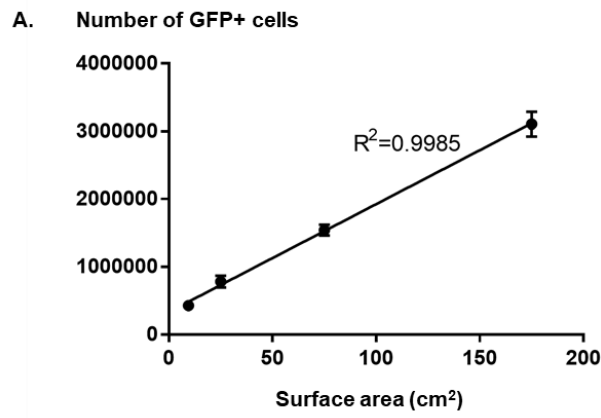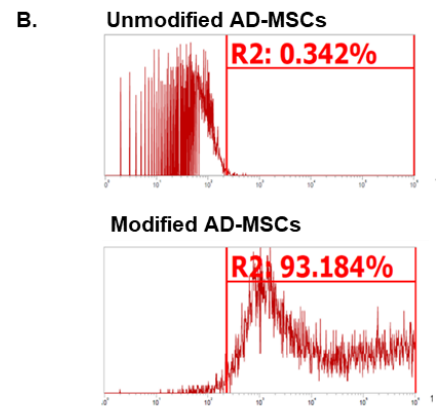

Supplement: Supplementary file 7 — Additional file 7. Scaling out MSC modification process on flat-bed culture. AD-MSCs were seeded in 6-well plate, T25, T75 and T175 flasks at 20,000/cm2. One day post seeding, cells were transfected with 150 ng PF463-CMV-GFP/ cm2 complexed at 1 μg pDNA to 3 μL PEI. Two-day post transfection, cells were harvested for analysis. Total number of cells harvested from each culture vessels were determined by the automated cell counter NC-3000. After which, the % of GFP+ cells measured with flow cytometry. a The scatter plot represents absolute number of GFP+ cells collected from each culture vessel; deduced from the readout of cell counting and flow cytometry of the GFP expression. Data of biological triplicates are expressed as mean + SD. b Representative images present the flow cytometry profile of unmodified and modified AD-MSCs harvested from T175 flasks. [file 13287_2020_1899_MOESM7_ESM.pdf]

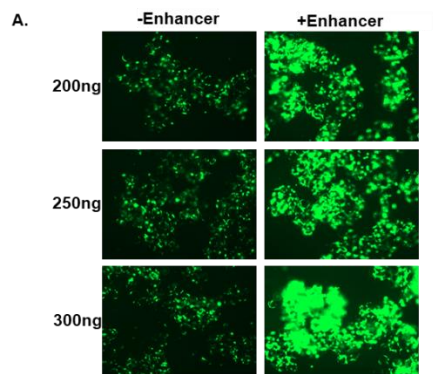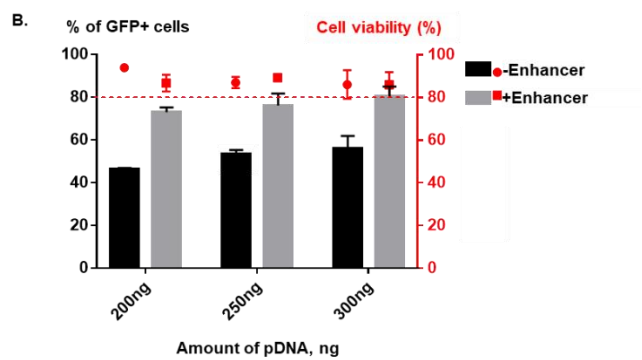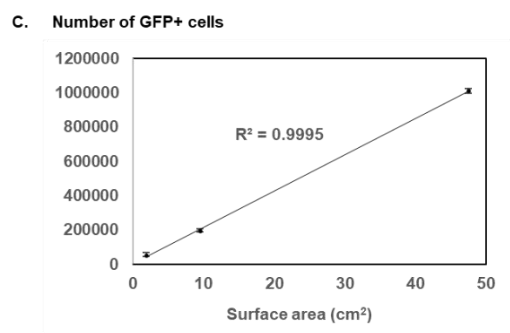

Supplement: Supplementary file 8 — Additional file 8. Scaling up MSC modification process on microcarrier culture. AD-MSCs were seeded at 28,000/cm2 on Cytodex® 3 microcarriers (total surface area of 1.9 cm2) in 24-well non-adherent plates, with agitation speed of 50 rpm for 24 h before transfection. Similar to flat-bed transfection, the polymer and DNA complex (at 200-300 ng of pDNA) were added to the cell culture using a dropwise manner after 15 min incubation. Cells were transfected in the presence or absence of the enhancer. Two-day post transfection, a the fluorescent images were captured at 10x magnification. Representative images are shown. b After which, cells were harvested through trypsinization. To separate the cells from microcarrier, the suspension cells were filtered through 70 μm cell strainer. The transfection efficiencies were determined through flow cytometry analysis. Cell viability based on PI exclusion was determined with NC-3000 automated cell counter. The combination chart presents % of GFP+ cells (graph bar) and cell viability (mark type) Data of biological triplicates are expressed as mean + SD. c For microcarriers with total surface area of 1.9 cm2 and 9.5 cm2, cells were transfected in 24-well non-adherent plates. To further scale up the transfection process, AD-MSCs were seeded on Cytodex® 3 (total surface area of 47.5 cm2) at 28,000 cells/cm2 in 125 mL Erlenmeyer flasks. One day later, cells were transfected in the presence of Enhancer. The amount of DNA was fixed at 150 ng/cm2 of microcarriers. Two-day post transfection, transfection efficiency was analysed. The scatter plot represents absolute number of GFP+ cells collected from each culture vessel; deduced from the readout of cell counting and flow cytometry analysis of the GFP expression. Data are expressed as mean + SD, n = 3. [file 13287_2020_1899_MOESM8_ESM.pdf]

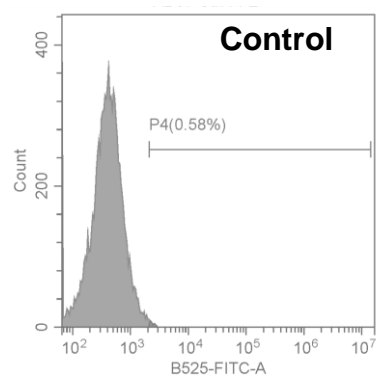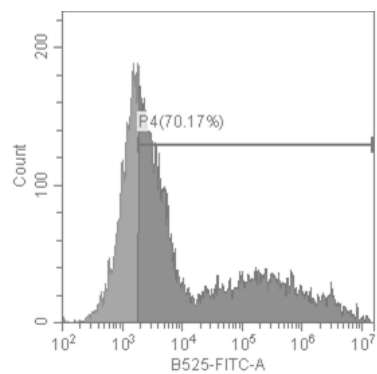

**-Enhancer**

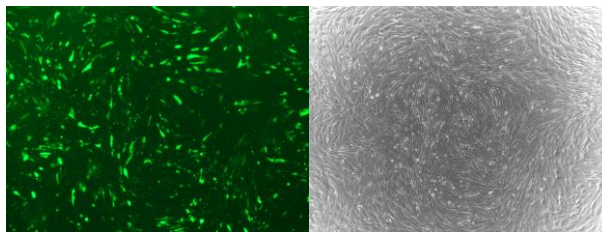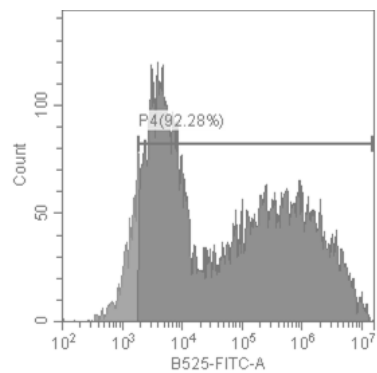

**+Enhancer**

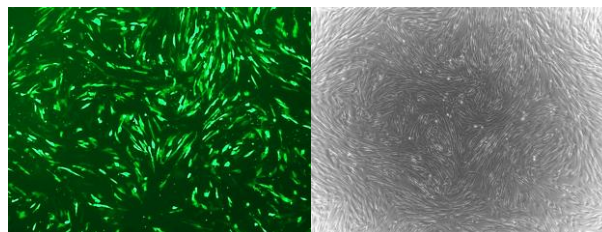

Supplement: Supplementary file 9 — Additional file 9. Representative images and FACS profile of AD-MSCs transfected with 300 ng of CD::UPRT::GFP plasmid, in the presence or absence of the Enhancer. [file 13287_2020_1899_MOESM9_ESM.pdf]

Osteogenic differentiation

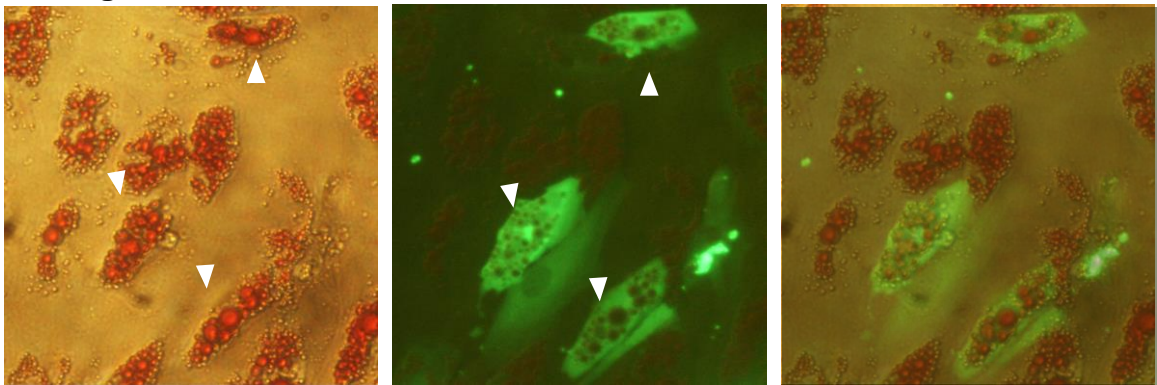

Overlay

Supplement: Supplementary file 10 — Additional file 10. Overlay of adipogenic differentiated AD-MSCs with GFP fluorescent image provides direct evidence of adipogenic differentiation of CD::UPRT::GFP expressing AD-MSCs. The images were captured at 20x magnification. Then, a representative set of images were cropped and enlarged. [file 13287_2020_1899_MOESM10_ESM.pdf]

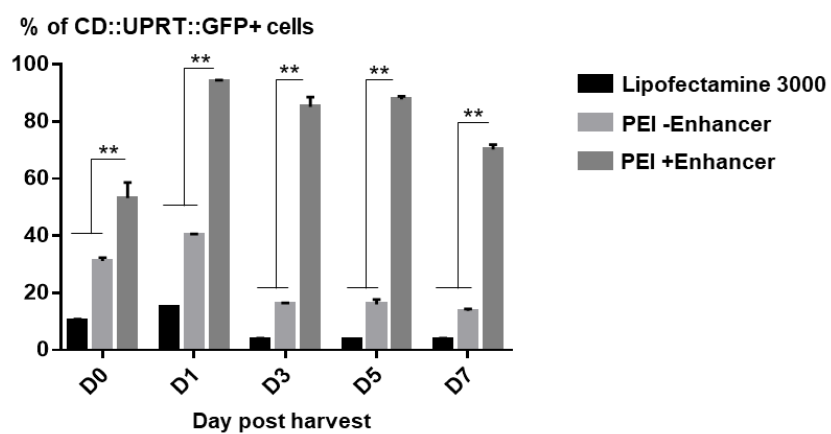

Supplement: Supplementary file 11 — Additional file 11. Prolonged expression of CDy::UPRT::GFP. One day post-transfection (D0), cells were harvested and seeded in 24-well plate for further incubation. Cells were harvested for CD::URPT::GFP expression analysis after 1, 3, 5, 7 days (D1, D3, D5, D7) of incubation. For each time point, cells were fixed with 4% PFA for batch analysis. The % of GFP+ cells were determined by flow cytometry analysis. Graph presents mean + SD (n = 3). Significant differences between PEI + Enhancer and other protocols were calculated using two tailed Student’s t-test. **, P < 0.005. [file 13287_2020_1899_MOESM11_ESM.pdf]

**A. Cell viability (%)**

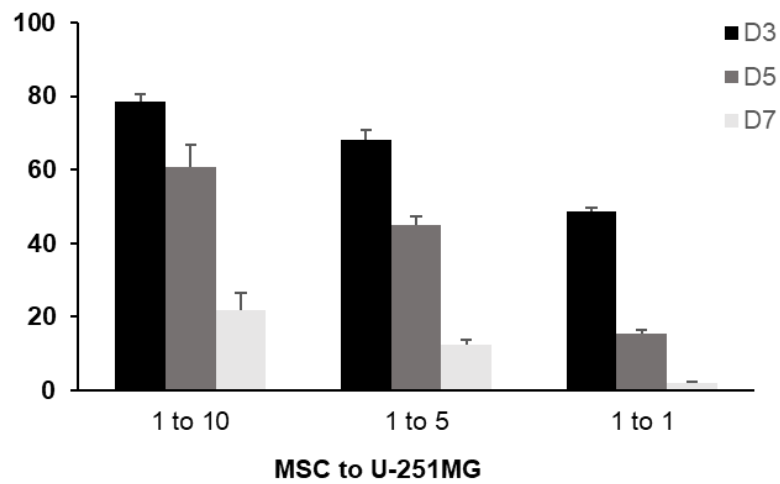

**B. Cell viability (%)**

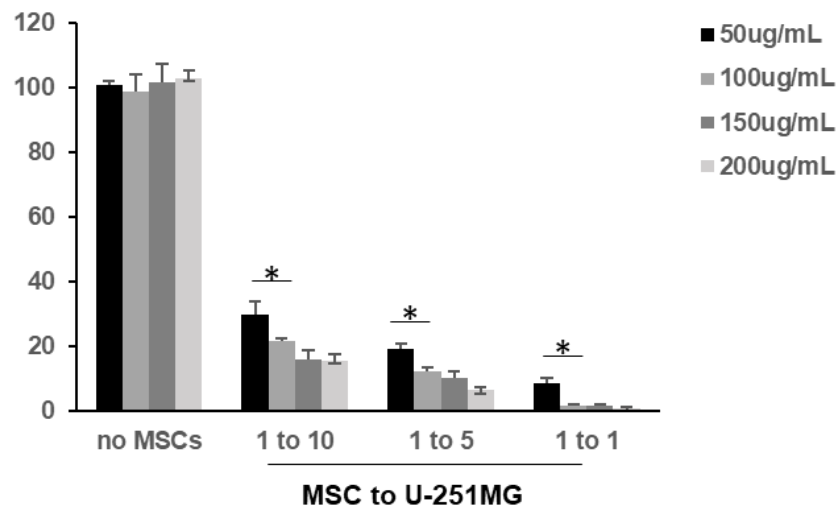

Supplement: Supplementary file 12 — Additional file 12. Coculture duration and 5FC concentration required for optimal in vitro cytotoxicity study. a CD::UPRT::GFP_AD-MSCs were cultured with U-251MG in low serum DMEM, in the presence or absence of 100 μg/mL 5FC. With pre-seeded U-251MG, the therapeutic cells were added at ratios of 1 CD::UPRT::GFP_AD-MSCs to 10, 5, 1 cancer cells. Three, five and seven days later (D3, D5, D7), cell viability in the treatment conditions was evaluated spectrophotometrically by MTS assay. Cell viability was defined as sample/control × 100%. Conditions without 5FC treatment served as controls for respective AD-MSCs to U-251MG ratios. Data of biological quadruplicates were expressed as mean + SD. b Similar study was performed to determine the 5FC concentration required for maximal cytotoxic effect. The coculture of CD::UPRT::GFP_AD-MSCs and U-251MG cells were treated with various concentration of 5FC for 7 days. At the end of the experiment, MTS assay was performed. Data of biological quadruplicates are expressed as mean + SD. Significant differences between 50 μg/mL of 5FC and other 5FC concentrations were calculated using two tailed Student’s t-test. *, P < 0.05. [file 13287_2020_1899_MOESM12_ESM.pdf]

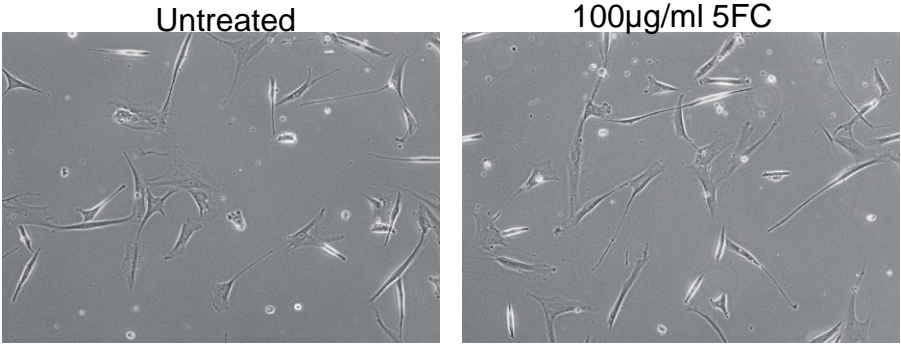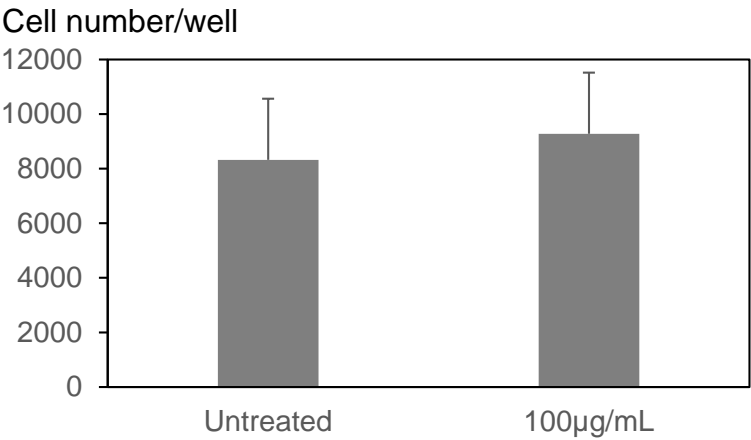

Supplement: Supplementary file 13 — Additional file 13. Unmodified AD-MSCs were seeded in 24-well plate at 2500/cm2. One day later, the culture media is replaced with low serum DMEM, in the presence or absence of 100 μg/mL 5FC. Cells were further incubated for 4 days. At the end of experiment, cells were trypsinised and subjected to cell count with the automated cell counter NC-3000. Graph bar presents the absolute cell number harvested from triplicates of each condition (mean + SD). [file 13287_2020_1899_MOESM13_ESM.pdf]

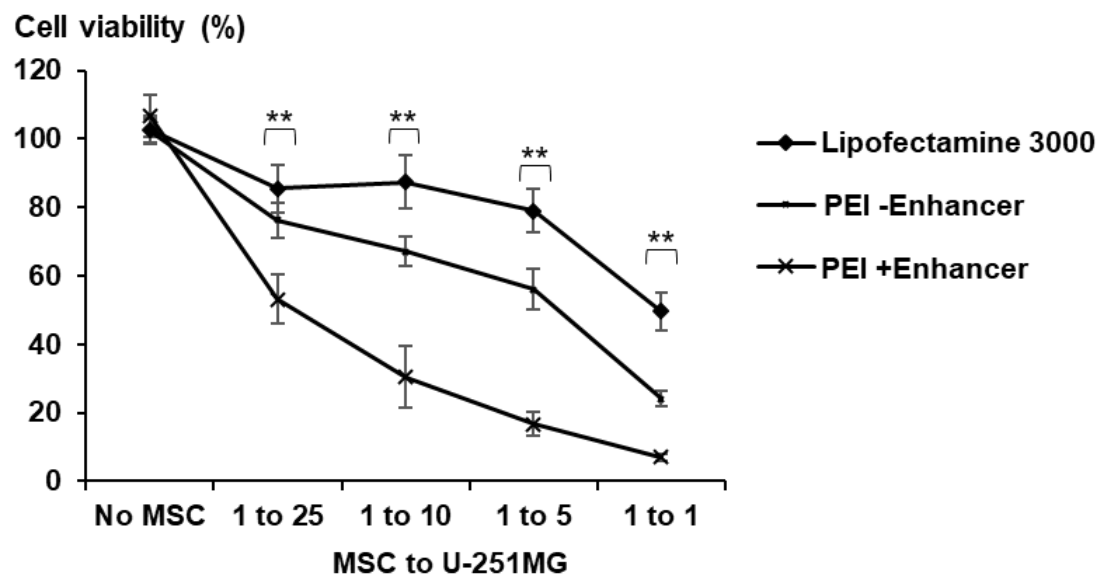

Supplement: Supplementary file 14 — Additional file 14. AD-MSCs generated with PEI plus Enhancers outperformed other methods. AD-MSCs were transfected with CD::UPRT::GFP plasmid using various protocols. Twenty-four hours post-transfection, CD::UPRT::GFP_AD-MSCs were cocultured with U-251MG in DMEM supplemented with 2% FBS, in the presence or absence of 100 μg/mL 5FC. The therapeutic cells were mixed at ratios of 1 CD::UPRT::GFP_AD-MSC to 25, 10, 5, 1 cancer cells. Seven days later, cell viability in the treatment conditions was evaluated spectrophotometrically by MTS assay. Cell viability was defined as sample/control × 100%. Conditions without 5FC treatment served as controls. No MSC condition suggests lack of 5FC cytotoxicity in cancer cells. Data presents mean + SD of the biological quadruplicates. Statistical differences between Lipofectamine 3000 and other methods were calculated using two tailed Student’s t-test. **, P < 0.005. [file 13287_2020_1899_MOESM14_ESM.pdf]

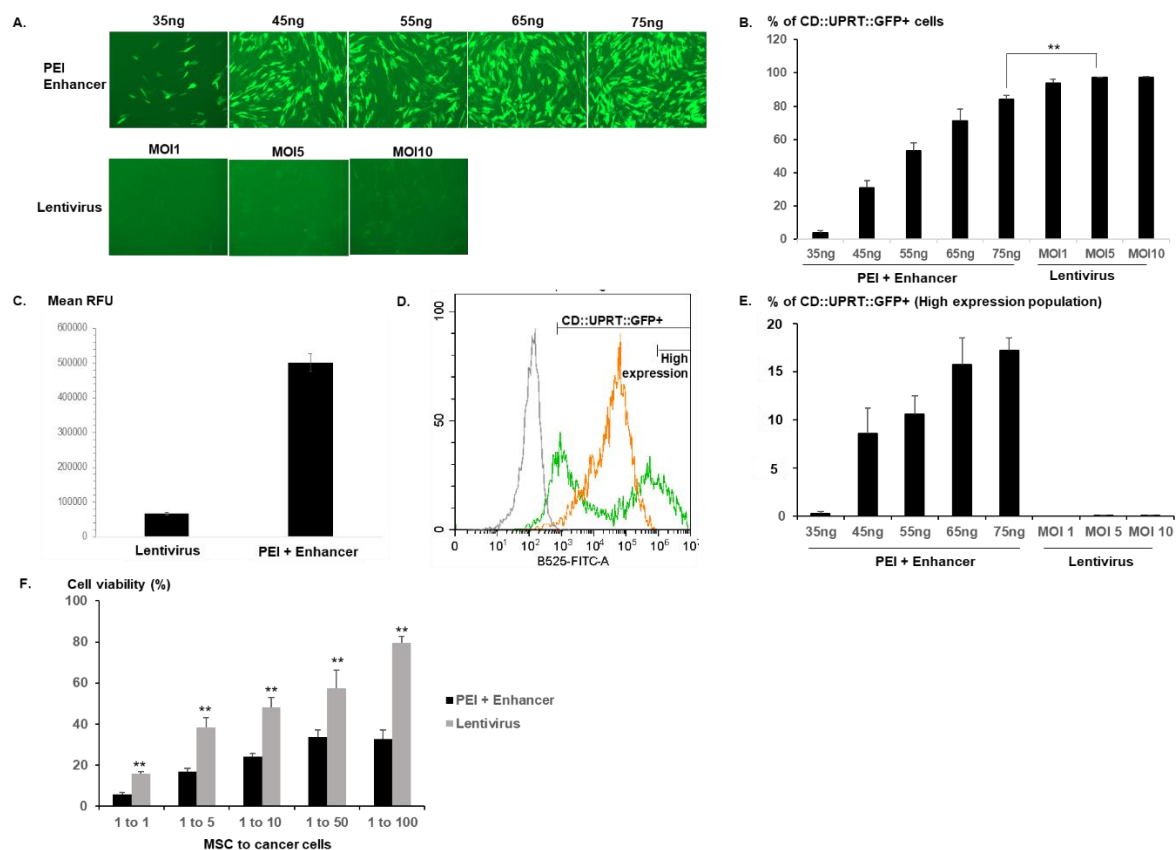

Supplement: Supplementary file 15 — Additional file 15. Non-viral modified AD-MSCs displayed higher anticancer potency. AD-MSCs were modified with PEI plus Enhancer and lentivirus carrying CD::UPRT::GFP at various pDNA amount and MOI, respectively. Two- or five-day post transfection or infection, a the fluorescent images were captured. Representative images are shown. b Then, cells were trypsinised, pelleted and resuspended in 1XPBS for flow cytometry analysis. Cell modification efficiency was calculated as % of CD::UPRT::GFP+ cells normalized to the total number of cells as quantified by FACS. c The mean RFU of AD-MSCs modified with lentivirus at MOI5 and non-viral method at 75 ng pDNA as measured by FACS is presented, n = 3. d The merged flow cytometry histogram displays the expression profile of the unmodified AD-MSCs (grey), AD-MSCs modified with 75 ng pDNA (Green) and lentivirus at MOI5 (orange). Population with FITC higher than 106 RFU is defined as population with high transgene expression. e The % of population with high transgene expression is presented with the bar graph, n = 3. f AD-MSCs modified transiently with PEI + Enhancer or stably with lentivirus were harvested for coculture with HT1080 cell line. One day later, cells were treated with 100 μg/mL of 5FC for 5 days. At the end of the experiment, MTS assay was performed to measure the cell viability. Bar graph represents mean of cell viability (%) ± SD, n = 4. Significant differences between PEI + Enhancer and Lentivirus were calculated using two tailed Student’s t-test. **, P < 0.005. [file 13287_2020_1899_MOESM15_ESM.pdf]

**-5FC**

**+5FC**

**U-251MG<sup>TMZR</sup>**

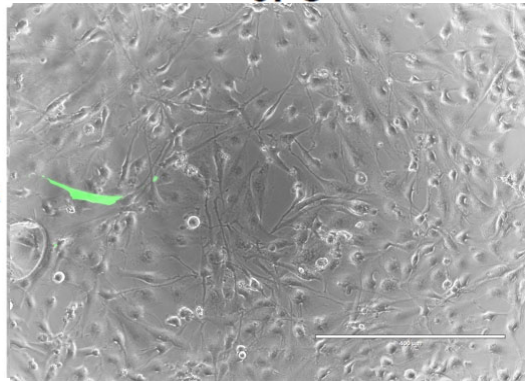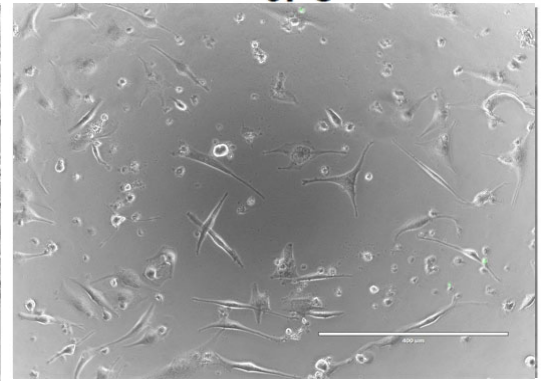

**U-87MG<sup>TMZR</sup>**

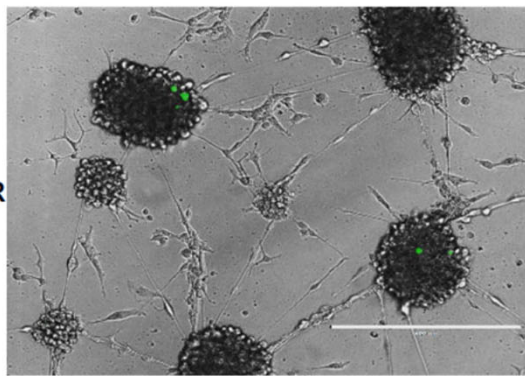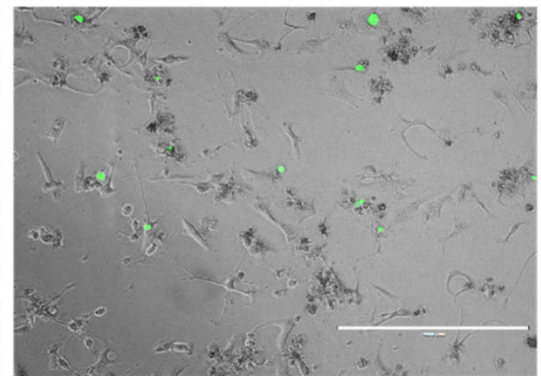

Supplement: Supplementary file 16 — Additional file 16. CD::UPRT::GFP_AD-MSCs were cocultured with U251MG™Z or U87MG™Z at the ratio of 1:10, in the presence or absence of 5FC. After seven days of incubation, images of the culture were captured. Representative images are presented. [file 13287_2020_1899_MOESM16_ESM.pdf]
