# Supplementary material for: A facile and scalable in production non-viral gene engineered mesenchymal stem cells for effective suppression of temozolomide-resistant (TMZR) glioblastoma growth
Source: Stem Cell Res Ther. 2020 Sep 11;11:391. doi: 10.1186/s13287-020-01899-x (PMC7488524; doi:10.1186/s13287-020-01899-x)
Supplement: Supplementary file 4 — Additional file 4. Gene expression z-score of patient derived cell lines for 5-FU pathway. DPYD, TYMS, UMPS, TYMP and ABCC5 expression levels are shown. The information was obtained from www.hgcc.se Dated: 3 Jun 2019. [file 13287_2020_1899_MOESM4_ESM.docx]

|  | **DPYD** | **TYMS** | **UMPS** | **ABCC5** |
| --- | --- | --- | --- | --- |
| U3008MG | -0.1 | -0.39 | -0.05 | 0.06 |
| U3020MG | 1.36 | -2.3 | -1.73 | -0.52 |
| U3054MG | 0.66 | 0.84 | -0.4 | 0.49 |
| U3117MG | 0.22 | 0.2 | -1.48 | -1.36 |
